# Divergence of gene regulation through chromosomal rearrangements

**DOI:** 10.1186/1471-2164-11-678

**Published:** 2010-11-30

**Authors:** Wolfgang Goettel, Joachim Messing

**Affiliations:** 1 Waksman Institute of Microbiology, Rutgers University, 190 Frelinghuysen Road, Piscataway, NJ 08854, USA

## Abstract

**Background:**

The molecular mechanisms that modify genome structures to give birth and death to alleles are still not well understood. To investigate the causative chromosomal rearrangements, we took advantage of the allelic diversity of the duplicated *p1 *and *p2 *genes in maize. Both genes encode a transcription factor involved in maysin synthesis, which confers resistance to corn earworm. However, *p1 *also controls accumulation of reddish pigments in floral tissues and has therefore acquired a new function after gene duplication. *p1 *alleles vary in their tissue-specific expression, which is indicated in their allele designation: the first suffix refers to red or white pericarp pigmentation and the second to red or white glume pigmentation.

**Results:**

Comparing chromosomal regions comprising *p1-ww[4Co63]*, *P1-rw1077 *and *P1-rr4B2 *alleles with that of the reference genome, *P1-wr[B73]*, enabled us to reconstruct additive events of transposition, chromosome breaks and repairs, and recombination that resulted in phenotypic variation and chimeric regulatory signals. The *p1-ww[4Co63] *null allele is probably derived from *P1-wr[B73] *by unequal crossover between large flanking sequences. A transposon insertion in a *P1-wr*-like allele and NHEJ (non-homologous end-joining) could have resulted in the formation of the *P1-rw1077 *allele. A second NHEJ event, followed by unequal crossover, probably led to the duplication of an enhancer region, creating the *P1-rr4B2 *allele. Moreover, a rather dynamic picture emerged in the use of polyadenylation signals by different *p1 *alleles. Interestingly, *p1 *alleles can be placed on both sides of a large retrotransposon cluster through recombination, while functional *p2 *alleles have only been found proximal to the cluster.

**Conclusions:**

Allelic diversity of the *p *locus exemplifies how gene duplications promote phenotypic variability through composite regulatory signals. Transposition events increase the level of genomic complexity based not only on insertions but also on excisions that cause DNA double-strand breaks and trigger illegitimate recombination.

## Background

An exciting challenge of biological research has been to understand phenotypic diversity within a species, which affects virtually every organ and cell type. In plants, this intraspecific diversity is often readily visible in the size, shape, color and number of flowers, fruits and seeds. Diversity can occur in every region of the gene, in coding regions or in regulatory sequences including upstream promoter and enhancer sequences, 5' and 3' UTRs and regulatory introns [1,2]. Changes in regulatory regions affecting allele expression and transcript amount can be simple, such as small and large indels, or more complex, such as transposon insertions and structural rearrangements. Molecular mechanisms responsible for the sequence modifications are replication errors, recombination and transposition. Although the majority of allelic variation is due to nucleotide polymorphisms, phenotypic differences can be caused by epigenetic modifications such as DNA methylation [3,4].

**Figure 1:**
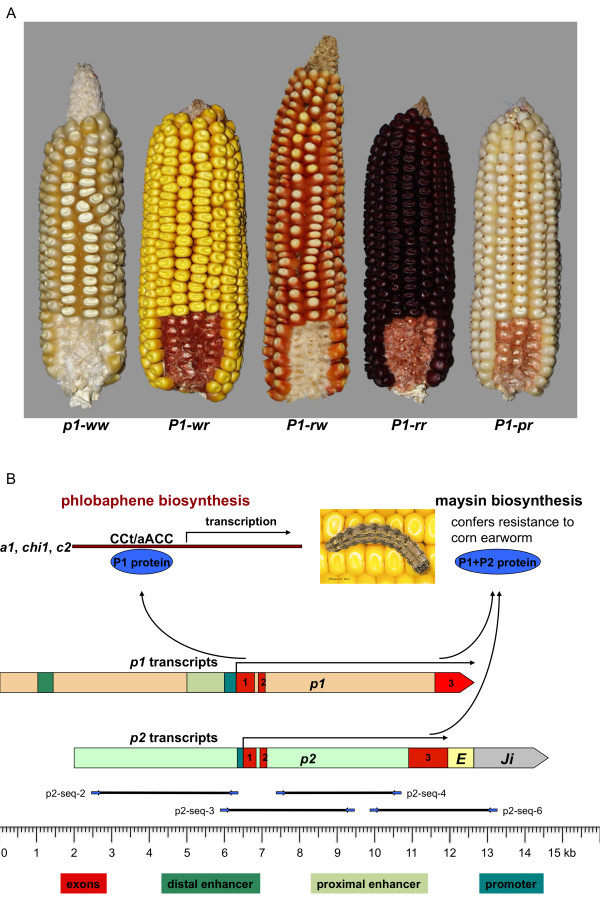
**Phenotype, structure and function of *p1 *and *p2 *alleles**. **(A) *p1 *alleles**. *p1 *alleles are phenotypically classified according to their pericarp (i.e. the outer layer of the kernel) and cob glume pigmentation. The first suffix of the allele designation refers to pericarp color (r for red, w for white or colorless, p for patterned), while the second suffix stands for glume color. **(B) Genomic structure of *p1 *and *p2 *alleles**. *p1 *alleles with the exception of *P1-rr *and *p2 *alleles contain three almost identical exons depicted in red. They also share a basal promoter sequence. However, enhancer regions that were identified in *P1-rr *are absent from *p2*. *p *regulatory elements are drawn in shades of green. The *p2 *coding sequence is flanked by fragments of *Eninu *(*E*) and *Ji *(*J*) retrotransposons. The P1 protein is a Myb-like transcriptional regulator of the phlobaphene biosynthesis pathway that activates transcription of the target genes *a1*, *chi1 *and *c2 *by binding to a CCt/aACC site. *p1 *is also a QTL for maysin accumulation. Maysin is a C-glycosyl flavone found in silk that confers resistance to corn. earworm (*Helicoverpa zea*, Boddie). While *p2 *does not control phlobaphene pigmentation, it is involved in maysin synthesis. PCR primers designed for *p2 *allele cloning and sequencing are indicated. Corn earworm image courtesy of Marlin E. Rice, Iowa State University Department of Entomology http://www.ent.iastate.edu/.

Sequence comparisons among inbred lines revealed that maize is a highly polymorphic species regarding genes and intergenic space [5]. Consequently, maize lends itself to studying the molecular basis of phenotypic variation. As an example for a detailed allelic analysis, we chose the *p1 *locus, which maps to the short arm of chromosome 1, for several reasons: 1) *p1 *produces a visible, quantitative phenotype in different tissues (Figure 1A). It encodes a R2R3 Myb-like transcription factor that activates the structural genes *c2*, *chi1 *and *a1 *of the phlobaphene biosynthesis pathway (Figure 1B) [6]. Phlobaphenes, which are reddish flavonoid pigments, accumulate in male and female floral organs. 2) The *p1 *gene is dispensable for the organism. Loss-of-function or change-of-function alleles will not be eliminated from the gene pool. 3) The *p1 *gene is characterized by its tremendous allelic diversity. More than hundred *p1 *alleles with distinct spatial and temporal expression pattern are reported although only few are molecularly defined [7]. 4) Approximately 2.75 million years ago, the *p1 *gene arose as a tandem duplication of the *p2 *gene [8]. If the *p2 *gene is the older of the two, it probably is the orthologous gene copy to the *p3 *gene on chromosome 9 because maize arose by allotetraploidization about 5 mya [9,10]. Therefore, we also refer to *p2 *as ortholog and *p1 *as paralog. The *p2 *gene is not involved in phlobaphene pigmentation, but like *p1*, is a QTL for maysin production (Figure 1B) [11].

Phlobaphene pigmentation is most readily visible in the pericarp, i.e. the outer layer of the kernel, and the cob glumes. Traditionally, *p1 *alleles are phenotypically categorized and named based on expression in these tissues. The *p1 *gene designation is followed by a two-letter suffix that refers to pericarp and cob color, respectively. For instance, *the P1-rr *allele exhibits red pericarp and red cob glume pigmentation while the *P1-rw *allele has red pericarp and white or colorless cob glumes (Figure 1A). Each phenotypic *p1 *group may consist of structurally very different alleles. Only few *p1 *alleles have been structurally determined of which only a small number has been completely or partially sequenced. *P1-rr4B2 *[12] and *P1-rw1077 *[13] are single copy genes that both were introgressed into the inbred line 4Co63. This inbred line contains a loss-of-function *p1-ww *allele. *P1-wr *in inbred line B73 is a multi-copy allele, consisting of 11 *P1-wr *tandem repeats that are flanked by *p2/p1 *and *p1/p2 *hybrid genes upstream and downstream of the cluster, respectively [10]. A large retroelement cluster is inserted in the 3' UTR of the *p1/p2 *hybrid gene.

The *p1-ww *alleles do not encode a functional P1 transcription factor; therefore pericarp as well as cob glumes are colorless (Figure 1A). While loss-of-function alleles often result in deleterious or even lethal conditions for the organism, non-functional *p1 *alleles do not cause any impairment that would reduce the fitness of the mutant plant. The *p1-ww *alleles can vary in origin and structure. Most of the structurally known *p1-ww *alleles are derived from *P1-rr *by transposon insertions and/or excisions. The *p1-ww1112 *null allele, for example, arose from a transposon-induced recombination event between the 5.2-kb direct repeats, which led to the deletion of the entire coding sequence [14]. However, the origin of *p1-ww *allele in the inbred line 4Co63 is not known, but *p1-ww[4Co63] *is often used in genetic crosses. Brink, for instance, introgressed more than 100 *p1 *alleles in the inbred line 4Co63 [7]. Knowledge of the *p1-ww[4Co63] *sequence could help clarify whether *p1-ww[4Co63] *is derived from *P1-rr *[12], *P1-wr *[10,15], *P1-rw *[13] or even a different *p1 *allele and provide further insights into other intermediates of chromosomal rearrangements.

To shed light on the origin of *p1 *allelic variability, we analyzed here three *p1 *alleles in their chromosomal context, namely *p1-ww[4Co63]*, *P1-rr4B2 *and *P1-rw1077*. First we resolve the structural organization of these *p1 *alleles and their corresponding *p2 *alleles on the single-nucleotide sequence level. Subsequently we compare their sequences also to the recently sequenced *P1-wr[B73] *cluster [10] to find large and small scale nucleotide polymorphisms that enable us to infer mechanisms for genome rearrangements. In particular, we focus on evolutionary changes in *p1 *alleles that occurred in the putative distal enhancer region and in the 3' UTRs.

## Results

### The structural organization of *p1-ww[4Co63] *and linked *p2 *gene

**Figure 2:**
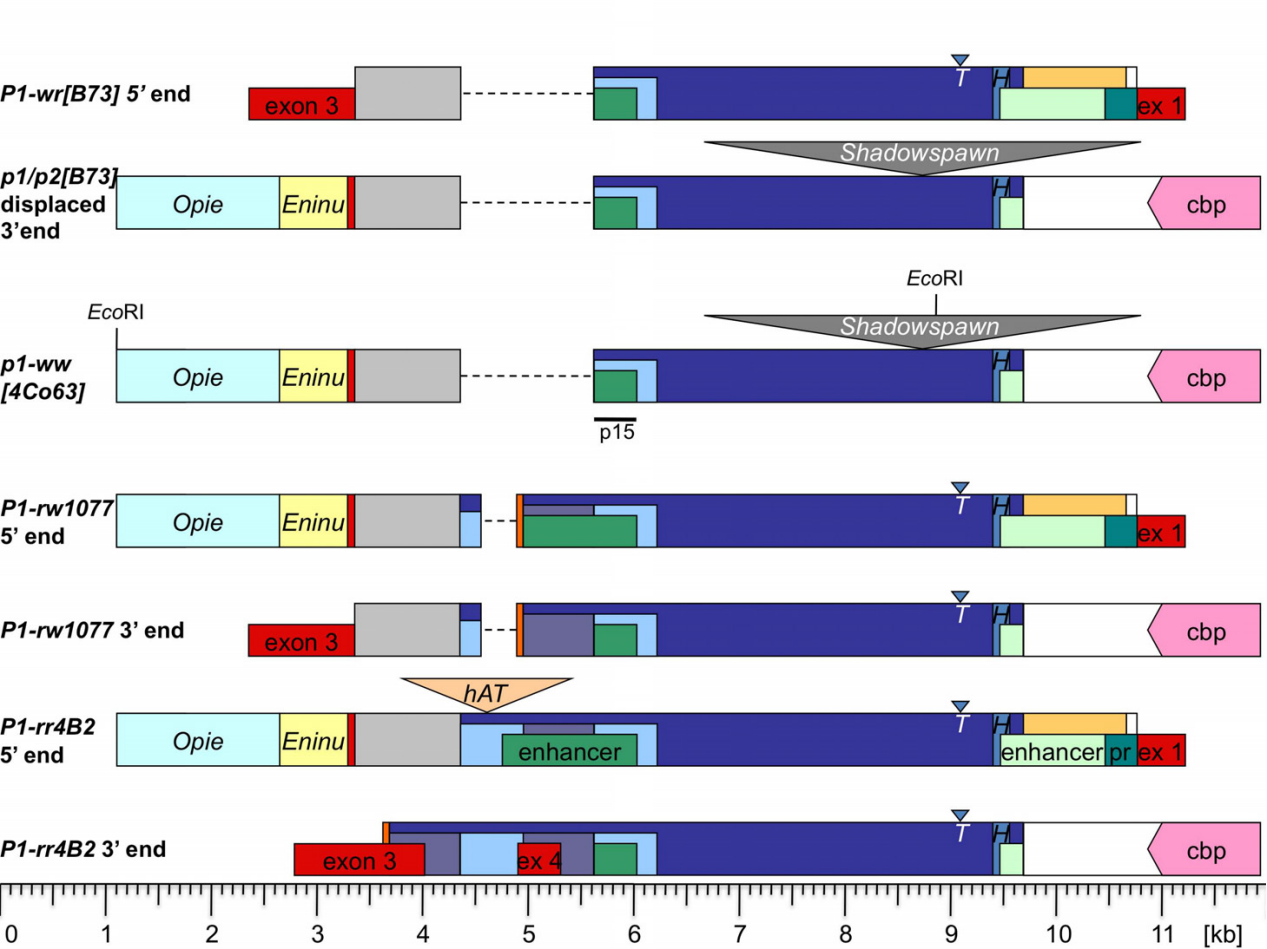
**Schematic alignment of *p *non-coding sequences**. *p *non-coding regions, which include regulatory elements (distal and proximal enhancer and promoter), can be located upstream, downstream or on both sides of *p *coding sequences. However, only *cis*-elements in the upstream non-coding regions have been shown to have regulatory function. *P1-wr[B73] *is the only *p1 *allele shown with a multi-gene structure, as indicated by exon 3 stemming from a neighboring *P1-wr[B73] *repeat. Due to the tandem array, each *P1-wr[B73] *coding region is flanked by this non-coding sequence. The *p1/p2[B73] *5' end (not shown) is identical to the *P1-wr[B73] *5' end. The *p1/p2[B73] *3' end is detached from the *p1/p2[B73] *coding region due to retrotransposon insertions. *Opie *(light turquoise rectangle), *Eninu *(light yellow rectangle) and the displaced former 3' UTR of *p1/p2[B73] *(red bar) are displayed. A shadowspawn retroelement (not drawn to scale) is inserted further downstream. *p1-ww[4Co63] *is virtually identical to the displaced *p1/p2[B73] *3' end. *P1-rw1077 *and *P1-rr *coding regions are bordered by large direct repeats (blue rectangles). Both 5' and 3' repeats are depicted with their adjacent exons indicating the omitted coding region. *p1-ww[4Co63] *and the *p1/p2[B73] *3' end are flanked by the same 5' sequences as *P1-rr *and *P1-rw1077 *(namely *Opie*, *Eninu *and the displaced 3' UTR). In addition, *p1-ww[4Co63] *and *p1/p2[B73] *share the 3' flanking sequences with *P1-rr *and *P1-rw1077 *as indicated by the downstream gene coding for a calmodulin-binding protein (cbp, pink pentagon). Notice that *P1-wr[B73]*, *P1-rw1077 *and *P1-rr *vary mainly in a region that contains a fragmented MULE insertion (purple rectangle) and a sequence specified by light blue rectangles, which is partially and completely duplicated in *P1-rw1077 *and *P1-rr*, respectively. The orange bar stands for the MULE TIR that is missing in the 5' *P1-rr *repeat. Regulatory elements, i.e. distal and proximal enhancer and basal promoter, depicted in shades of green, were only determined for *P1-rr*. In other *p *genes or alleles, green rectangles merely refer to sequence similarity with *P1-rr*. Functional homology has not been investigated. The dark yellow rectangle represents a further *Mu*-like transposon that overlaps with the proximal enhancer and promoter region. A *Heartbreaker *MITE (blue bar) is part of the proximal enhancer region. Transposable elements of various families are shown as triangles above the schematic sequences. Notice the missing *Tourist *MITE in *p1-ww[4Co63] *and the displaced 3' end of *p1/p2[B73]*. Exons (red rectangles) are added with the intention to facilitate orientation. The *Eco*RI cloning sites and the hybridization site of probe 15 used to screen the lambda library for *p1-ww[4Co63] *sequencing are marked.

A partial genomic lambda library was constructed using *Eco*RI-digested 4Co63 DNA. Filters were screened with the probe p15 (Figure 2), which hybridizes to a distal enhancer region thought to be present in all *p1 *alleles at the time of *p1-ww[4Co63] *cloning. A lambda clone containing 11,073-bp genomic DNA was isolated and sequenced [GenBank:HM454274]. Interestingly, this sequence is 99.7% identical to the displaced *p1/p2[B73] *3' UTR and its 3' intergenic region, starting from an *Eco*RI recognition sequence in the retrotransposon *Opie*, and ending in an *Eco*RI site in the retroelement *Shadowspawn *(Figure 2).

The extensive similarity with the *p1/p2[B73] *3' end and intergenic region together with the identical *Shadowspawn *insertion suggests that both sequences continue to be similar past the end of the lambda clone. To confirm this assumption, we extended the sequence by genomic PCR from the *Shadowspawn *element to neighboring genes that are unrelated to *p *and therefore do not participate in potential *p *recombination events. PCR primer pairs were designed based on the equivalent *P1-wr[B73] *cluster, and PCR products were cloned and sequenced [GenBank:HM454275]. The analysis of 6,587 bp revealed that 4Co63 and B73 are virtually identical in this sequence; they consist of the 3' end of the *Shadowspawn *retroelement, a gene encoding a calmodulin-binding protein, part of a gene encoding a protein of unknown function, and intergenic regions (Figure 2). The calmodulin-binding protein, which in 4Co63 measures 361 aa, is 7 aa larger than in B73 and also contains two amino acid substitutions due to 3 indels and 10 SNPs. Based on maize and other EST data, this gene is transcribed and is very conserved in grass species such as rice, sorghum and barley.

**Figure 3:**
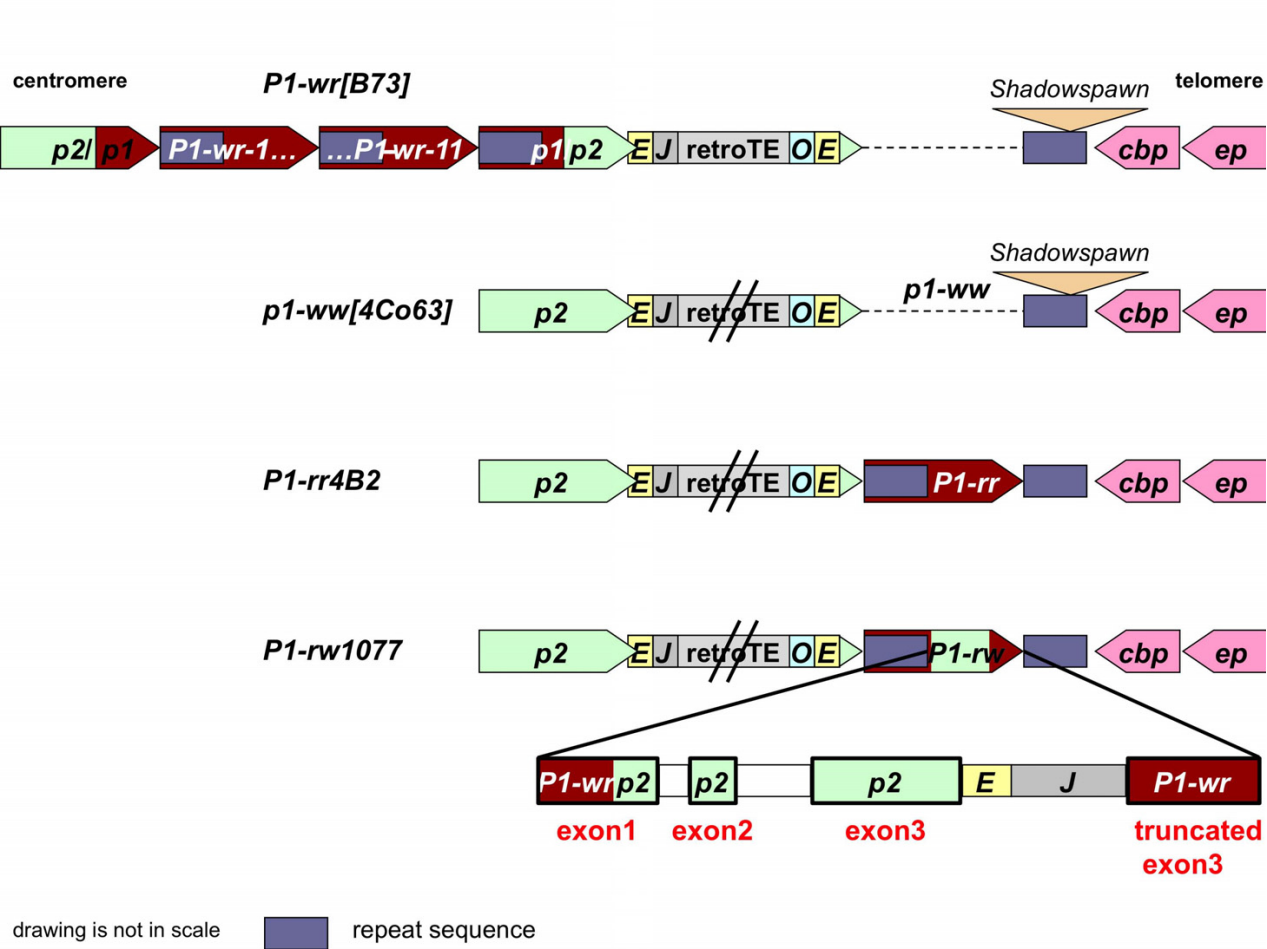
**Schematic alignment of *p1 *and *p2 *alleles**. *P1-wr[B73]*, *p1-ww[4Co63]*, *P1-rw1077 *and *P1-rr4B2 *alleles are represented as dark red and their corresponding *p2 *alleles as light green pentagons with their apex pointing in the direction of transcription. The retrotransposon cluster downstream of *p2 *that was only entirely sequenced in *P1-wr[B73] *is 68 kb in size. In all remaining lines only the end sequences of the cluster that consist of *Eninu *(*E*), *Ji *(*J*) and *Opie *(*O*) retroelement fragments were determined. Downstream flanking genes, which encode a calmodulin-binding protein (here labeled as *cbp*) and an expressed protein (*ep*), are illustrated as pink pentagons. Both genes are conserved in grasses and arranged in opposite transcriptional orientation to *p *alleles. Purple rectangles refer to a sequence that most likely originated as the 3' intergenic region of an ancestral *p *gene. Due to the duplication event that gave rise to *p1 *and *p2*, this region also became present upstream of *p1 *alleles (see Figure 2 for details). The triangles on top of some purple rectangles stand for retrotransposon (*Shadowspawn*) insertions. The coding regions of *p2/p1[B73], p1/p2[B73] *and *P1-rw1077 *consists of *p2 *and *p1 *sequences. In *P1-rw1077*, the 5' end of exon 1 can be attributed to *p1 *while the 3' end of exon 1, exon 2 and 3 and flanking retrotransposon sequences are derived from *p2 *[13]. Interestingly, the retroelements are followed by a truncated *P1-wr[B73] *exon 3. Note that the drawing is not in scale.

Although we could not find any remnants of *p1 *in the 3' flanking regions, we also needed to extend the analysis to the 5' flanking regions. We knew that a functional *p2 *gene, as visualized in silk browning reactions, still had to be present. The browning reaction of freshly cut back silk correlates with silk maysin concentration and is therefore induced by both *p *genes [16]. To extend the sequences of *p1-ww[4Co63] *up to the *p2 *gene, we used a genomic PCR approach by taking advantage of existing *p2*, *p2/p1[B73] *and *p1/p2[B73] *sequences for the primer design. We sequenced a total of 10,753 bp that include the complete *p2 *and flanking sequences [GenBank:HM454271] (Figure 3). No *p1 *fragments or traces were detected. The *p2[4Co63] *and *p2[p1ww1112] *alleles with their flanking sequences are 94.56% identical. They differ in 57 SNPs and multiple indels, of which the largest consists of a *Heartbreaker *MITE insertion of 317 bp in the second intron. Because the 1,008 bp coding sequences of the two duplicated genes only vary by one synonymous substitution each in exon 2 and 3, their deduced P2 protein sequences of 335 aa are identical (Additional file 1: Supplemental Figure S1). RT-PCR experiments confirm that *p2[4Co63] *is expressed in silk tissue as predicted by the silk browning reaction (data not shown).

**Figure 4:**
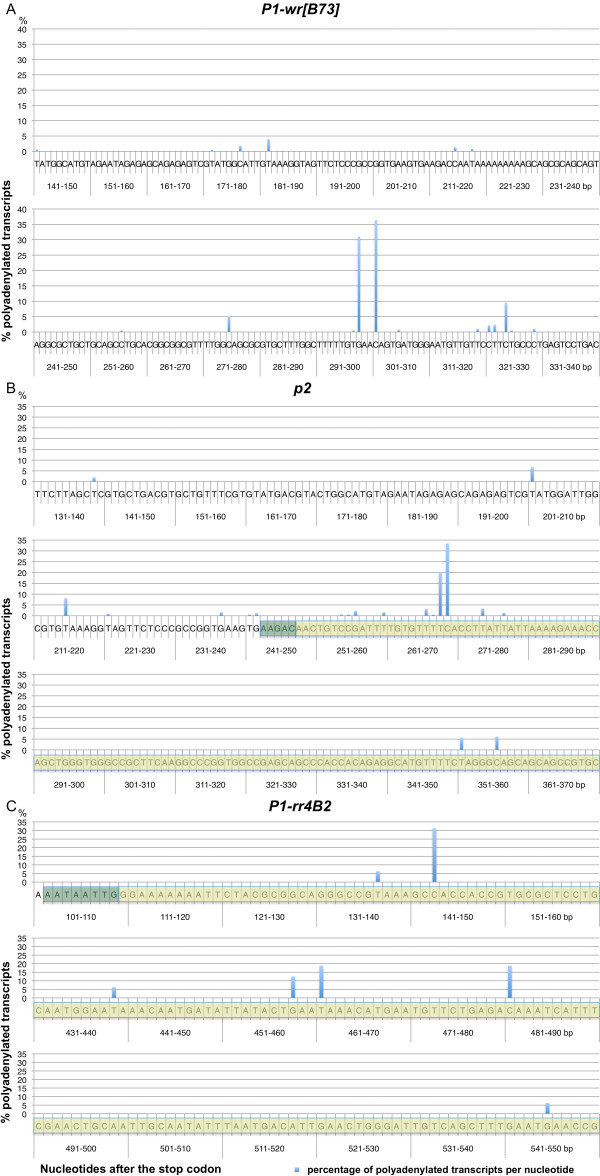
**Polyadenylation sites of *P1-wr[B73]*, *P1-rr4B2 *and *p2***. Figures A to C display the partial 3' UTR sequences of *P1-wr*, *p2 *and *P1-rr*, respectively. Numbers on the x-axis refer to the nucleotide position after the stop codon. The y-axis reflects the percentage of transcripts that were polyadenylated at each nucleotide. (A) *P1-wr[B73] *polyadenylation sites. 18 polyadenylation sites are shown for *P1-wr *transcripts isolated from pericarp tissue. The most frequently used polyadenylation sites are 298 nt and 301 nt downstream of the stop codon. (B) Polyadenylation sites of *p2 *are located in a flanking LTR region. The sequence highlighted in yellow belongs to an *Eninu *retrotransposon that inserted 1.38 mya in *p2*. The target site duplication (TSD) AAGAC upon insertion is highlighted in green. The first and second bp of the LTR are mutated from TGCTGT to AACTGT. The majority of transcripts are polyadenylated within the LTR sequence. (C) Polyadenylation sites of *P1-rr4B2 *are located in a flanking MULE sequence. The MULE sequence is highlighted in yellow and the potential TSD in green. Note the sequence gap 161-430 bp after the stop codon, which does not contain any polyadenylation sites. All *P1-rr4B2 *transcripts are polyadenylated within the transposon.

The sequence 246 bp downstream of the *p2 *stop codon is composed of a partial *Eninu *retroelement of 540 bp followed by a *Ji *retrotransposon, which covers the remaining 1,322 bp of the available sequence (Figure 3 and 4B). The 4Co63 *p2 *allele differs from the B73 *p2 *sequences extracted from the *p2/p1[B73] *and *p1/p2[B73] *alleles in many SNPs and indels including transposon insertions, suggesting that *p1-ww[4Co63] *may not have arisen from *P1-wr[B73] *by recombination events in a direct lineage.

Interestingly, the retrotransposons at the 3' end of *p2*, namely *Eninu *and *Ji*, and at the 5' end of the above described "*p1-ww*" lambda clone, namely *Opie *and *Eninu*, are identical to the retroelement cluster of *P1-wr[B73] *in sequence, insertion site and consequently target site duplications. Although we did not clone and sequence the complete retroelement cluster in *p1-ww[4Co63] *it is most likely that both clusters in 4Co63 and B73 are identical, at least in their initial transposition of *Eninu *and their nested insertions of *Ji *and *Opie *(Figure 3).

In brief, whereas *p2 *is present and functional in the 4Co63 inbred line, *p1 *coding and regulatory sequences are missing with the exception of the distal enhancer region. The structure of the *p1-ww[4Co63] *allele does not unambiguously point to a single known *p1 *allele where *p1-ww[4Co63] *is derived from, although, mechanistically, unequal crossing over between flanking sequences of the *p1 *gene could have been involved as discussed below.

### The structural organization of *P1-rr4B2 *and linked *p2 *gene

How does the sequence arrangement of *P1-wr[B73] *and *p1-ww[4Co63] *including their flanking genes compare to *P1-rr4B2*, a *p1 *single-copy allele that produces red pericarp and red glumes? *P1-rr4B2 *contains two large repeats flanking the coding sequence, which are about 5.2 kb in size [6,12,17]. Interestingly, the sequence upstream of the 5' large repeat contains fragments of *Opie *and *Eninu *retroelements inserted in the same position as in *p1-ww[4Co63] *and *p1/p2[B73] *as described above (Figures 2 and 3). Likewise, *Eninu *is bordered by the detached *p *3' UTR sequence of 78 bp. Subsequently, *P1-rr *is highly similar to a single *P1-wr[B73] *copy with few exceptions: the upstream regulatory region is more complex in *P1-rr *than in *P1-wr[B73] *(Figure 2) and both sequences diverge shortly after the stop codon (see below). By sequencing two plasmids, SA206 and PA103, which contain the 3' large repeat and are derived from lambda clones used for the isolation of *P1-rr *5' and coding sequences [18] (see Methods), we extended our *P1-rr *sequence analysis by 8,923 bp past the 3' large repeat and into flanking genes [GenBank:HM454276]. By aligning both large repeats we found 14 polymorphisms including the insertion of a transposable element of 1,616 bp in the 5' repeat (Figure 2). This element is flanked by 8-bp direct repeats (CCAGTGAG), which is typical for transposons of the *hAT *superfamily. The 3' large repeat following the upstream regulatory sequence resembles *p1-ww[4Co63] *but does not contain the *Shadowspawn *retrotransposon insertion (Figures 2 and 3). Furthermore, the final 4,341 bp of the plasmid insert, not related to *P1-rr*, are highly similar to the equivalent *p1-ww[4Co63] *and *P1-wr[B73] *sequences (Figures 2 and 3). The 3' flanking sequence contains one complete gene and one partial gene in opposite transcriptional orientation compared to *P1-rr*. The first gene, which is separated from *P1-rr *by 1,175 bp (measured from the end of the 3' *P1-rr *repeat to the stop codon), encodes the 4Co63-type calmodulin-binding protein consisting of 361 amino acids. No more than 609 bp of intergenic sequence divide the first from the second gene, of which only the final two exons are present in the plasmid clone.

The *P1-rr *sequence analysis revealed that *P1-rr *is located between the retroelement cluster and the gene encoding a calmodulin-binding protein. Most interestingly, the corresponding site in *p1-ww[4Co63] *and *P1-wr[B73] *is empty, i.e. this region does not contain a *p1 *gene copy (Figure 3). Based on the first maize *p2 *allele that was isolated from a line which contains the *p1-ww1112 *allele [8] we assume that a functional *p2 *allele of *P1-rr4B2 *is present upstream of the retroelement cluster because *p1-ww1112 *and *P1-rr4B2 *are both derived from the same allele. Furthermore, the *p2[p1-ww1112] *allele ends in *Eninu *and *Ji *retroelement fragments exactly like *p2[4Co63] *and *p1/p2[B73] *suggesting structural similarity among these alleles. Therefore, we decided to extend our sequence analysis to the *p2 *allele that is linked to *P1-rr4B2*. We used the same genomic PCR strategy as described above to clone and sequence 10,423 bp of *p2[P1-rr4B2] *[GenBank:HM454272]. Indeed, the alignment of *p2[P1-rr4B2] *with *p2[p1-ww1112] *showed no SNPs but only four 1-bp indels that are not part of exons or putative regulatory sequences. Hence both *p2 *alleles are coding for an identical P2 protein (Additional file 1: Supplemental Figure S1). As expected, *p2[P1-rr4B2] *is also flanked by *Eninu *and *Ji *retroelement sequences. Introgression of *P1-rr4B2 *in 4Co63 probably included *p2 *as well because *p2[P1-rr4B2] *differs from *p2[4Co63]*.

### The structural organization of *P1-rw1077 *and linked *p2 *gene

The *P1-rw *allele specifies red pericarp and colorless cob glumes (Figure 1A). In general, the structure of *P1-rw1077 *resembles *P1-rr4B2 *[13]. *P1-rw1077 *is a single-copy gene, which consists of a coding region flanked by two 6.3-kb direct repeats (Figure 2). The coding sequence of *P1-rw1077 *is chimeric in nature. While the 5' UTR is similar to *p1*, the remaining coding region and adjacent *Eninu *and *Ji *retroelements (spanning about 6.9 kb) are *p2*-like (Figure 3) [13]. Sequence alignments establish that the *p2 *fragment is more closely related to *p2[P1-rr4B2]*/*[p1-ww1112] *than to *p2[4Co63]*. Interestingly, the *Ji *retrotransposon is followed by a truncated *P1-wr*-like exon, which is not included in the *P1-rw1077 *transcript. This organization of sequences suggests that *P1-rw1077 *originated from a gene conversion event between *p1 *and *p2 *[13]. The *P1-rw1077 *sequence upstream of the 5' large repeat is very similar to the corresponding *P1-rr4B2 *region, suggesting that both alleles occupy the same chromosomal location. We confirmed this by PCR-amplification and sequencing of a 1,651-bp fragment that connects the 3' large repeat of *P1-rw1077 *with the gene encoding the calmodulin-binding protein. *P1-rw1077 *was introgressed in 4Co63, and indeed the 3' end of the intergenic region between the 3' large repeat and the neighboring gene is indistinguishable from 4Co63. Since *P1-rw1077 *and *P1-rr4B2 *occupy the same chromosomal position we wanted to find out whether the similarity extends to the region upstream of the retrotransposon cluster (Figure 3). We performed genomic PCR as described above to amplify and subsequently sequence 11,313 bp [GenBank:HM454273] that are 99.8% identical to the *p2[4Co63] *sequence. The 18 SNPs and 3 short indels, which are distributed over a consensus sequence of 10,703 bp, are not included in the *p2[P1-rw1077] *coding sequence and consequently do not alter the P2 protein sequence (Additional file 1: Supplemental Figure S1). The polymorphisms between *p2[P1-rw1077] *and other *p2 *alleles suggest that this *p2 *sequence was introgressed together with *P1-rw1077 *into the 4Co63 background. This implies that the *p2 *part of *P1-rw1077*, which is *p2[P1-rr4B2]*-like, is derived from a *p2 *source other than *p2[P1-rw1077]*. The *p2[P1-rw1077] *3' sequence is also flanked by *Eninu *and *Ji *retroelement fragments, linking *p2[P1-rw1077] *to *P1-rw1077 *across the retrotransposon cluster (Figure 3).

In summary, while *p1 *alleles can be located on both sides of the retroelement cluster (Figure 3), complete *p2 *alleles have so far only been found upstream of the retroelement cluster.

### Evolution of a putative distal enhancer by non-homologous end-joining and transposition

Because all known *p1 *alleles produce almost identical P1 proteins (Additional file 1: Supplemental Figure S1), differential expression of *p1 *alleles could have evolved through changes in regulatory sequences, which control time-and tissue-specific *p1 *expression [17]. Sequences containing regulatory elements are only determined for *P1-rr *[19,20], but based on sequence similarities have likely the same function in other *p1 *alleles as well. While all known *p1 *alleles share the *P1-rr *promoter and proximal enhancer sequences, they differ in the sequence arrangement that contains the distal *P1-rr *enhancer. Comparing putative distal enhancer regions of *p1 *alleles reveals that the single *P1-wr[B73] *gene carries the simplest and therefore possibly the most ancestral form, which is confirmed by the presence of an almost identical enhancer region at the 3' intergenic region of the *p2 *gene in a wild relative of maize (Teosinte accession *Zea mays *ssp. *parviglumis*) [8]. Complexity of this chromosomal region increased with *P1-rw1077 *and then *P1-rr4B2*. Therefore, we can use the changes in sequence organization to explain the origin of the *P1-rw1077 *and *P1-rr4B2 *enhancer region within the *P1-wr *repeat context, where the 3' end of one copy equals the 5' end of the downstream copy.

**Figure 5:**
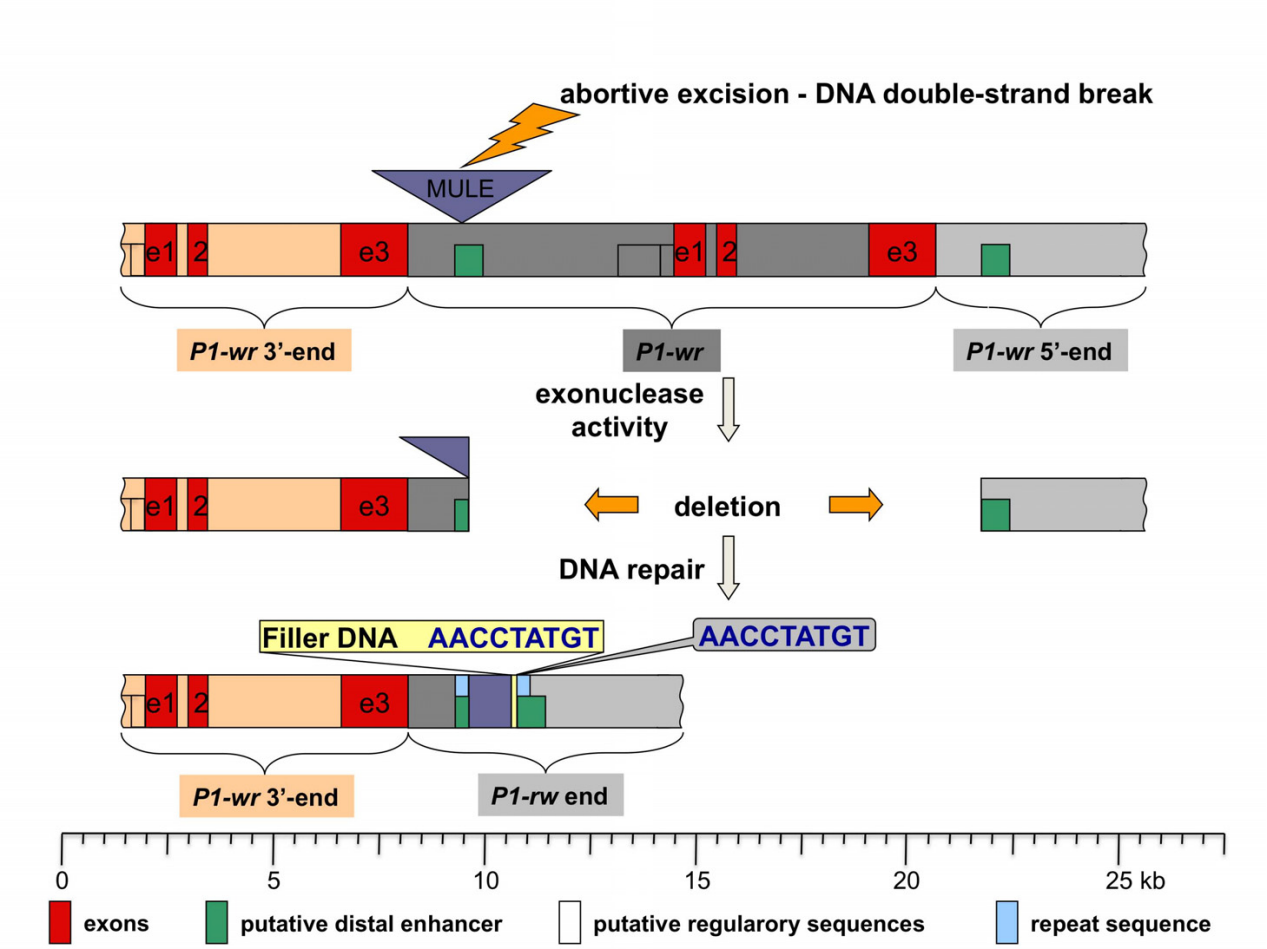
**Model for the origin of the *P1-rw1077 *enhancer region by non-homologous end-joining**. The bottom bar illustrates the *P1-rw1077 *end adjacent to a *P1-wr *repeat. The top bar represents schematically a full length *P1-wr *repeat flanked by partial *P1-wr *copies on both sides (drawn as dark grey, tan and light grey rectangles, respectively). The green rectangle indicates part of a sequence that was proven to have enhancer function in *P1-rr*. A model demonstrating the progression from a *P1-wr *sequence to a unique *P1-rw1077 *enhancer structure is briefly outlined. A DNA double-strand break was initiated by the excision of a *Mu*-like transposable element (purple triangle) and the resulting gap was expanded by exonuclease activity. DNA repair was accomplished by non-homologous end-joining as implied by the presence of filler DNA (yellow rectangle). The filler DNA AACCTATGT is derived from a sequence close to the deletion end point (see light grey balloon). The neighboring 725 bp (purple rectangle) originated from the excised MULE. The light blue rectangles specify sequences that are duplicated due to the deletion of less than a full-length *P1-wr *repeat. Note that the terminal *P1-wr *copy only contains the 5' end and is similar to the 3' large repeat of *P1-rr *and *P1-rw*, which does not have any gene function and therefore should be considered as intergenic region. Due to the unknown origin of *P1-rw1077 *and *P1-rr*, we use the designation *P1-wr *also for *P1-wr-*like alleles that share regulatory and coding regions with *P1-wr[B73]*.

*P1-rw1077 *is a complex allele that must have been shaped by multiple recombination events (Figure 3) [13]. Interestingly, the sequence following the *p2 *portion resembles the junction of two *P1-wr[B73] *copies in a head-to-tail arrangement, suggesting that *P1-rw1077 *arose from *P1-wr*-like tandem repeats. The sequence similarity between *P1-rw1077 *and *P1-wr[B73] *starts with the truncated exon 3. *P1-rw1077 *and *P1-wr[B73] *are identical until they diverge 1,001 bp after the truncated *P1-rw1077 *exon 3. The next 734 bp of *P1-rw *are of mixed origin and mostly unrelated to *P1-wr*. *P1-rw *continues its homology with *P1-wr *3' of the 734-bp insertion, but not at the sequence where both alleles deviate from each other. Instead, the *P1-rw *sequence downstream of the insertion is identical to the region of *P1-wr *upstream of the insertion, i.e. the insertion is flanked by a 203-bp repeat sequence. The sequence after the point of divergence originated from an unknown *Mu*-like transposable element (MULE) in reverse orientation (Additional file 2: Supplemental Figure S2). Based on BLASTN searches, the sequence consists of two MULE fragments that in a putative autonomous MULE would be separated by approximately 444 bp (for additional information on this new MULE family in the maize B73 genome see Additional file 3). While the initial 279 bp, starting with the TIR (GGAAAAAATT...), are derived from the MULE 3' end, the remaining 446-bp fragment stems from a sequence partially encoding the C-terminus of the MULE transposase. The final 9 bp (AACCTATGT) of the 734-bp insertion may represent filler DNA (see bottom panel of Figure 5). The 9-bp fragment is identical to a *P1-wr[B73] *sequence, which is located 27 bp downstream of the point of *P1-rw1077 *and *P1-wr[B73] *alignment. Filler DNA, which is usually found at repair sites of DNA double-strand breaks can be simple as described here or complex, consisting of a patchwork of multiple sequences. Filler DNA is associated with non-homologous end-joining and is usually derived from nearby sequences of either end of the break [21-23] (for additional information on the mechanism of NHEJ see Additional file 4).

**Figure 6:**
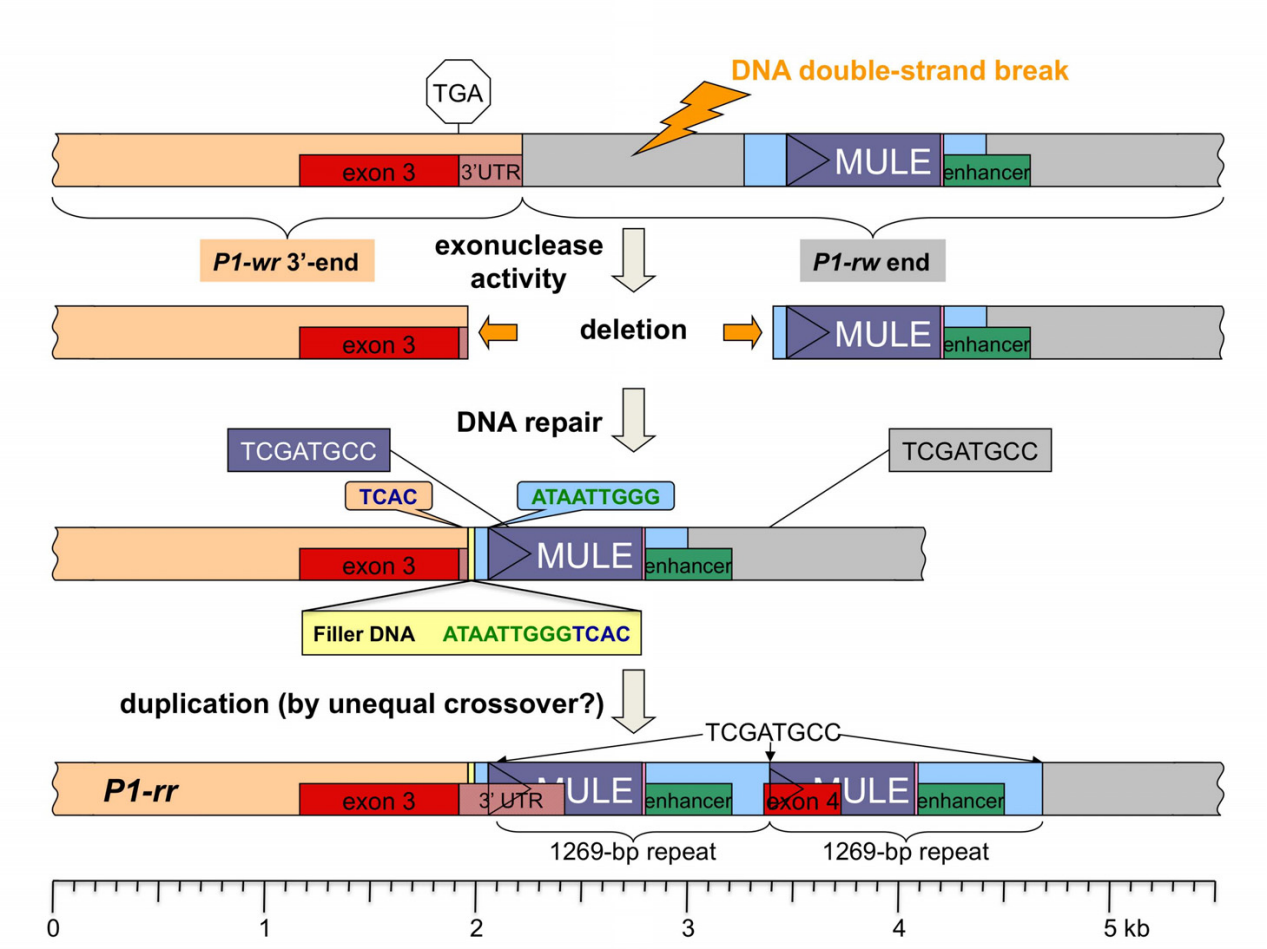
**Model for the origin of the unique *P1-rr *3' end and enhancer region by non-homologous end-joining and unequal crossover**. The bottom bar represents the unique *P1-rr *3' end with tandem direct repeats. The top bar shows schematically the junction sequence of two *P1-wr *repeats drawn as tan and grey rectangles. The *P1-wr *copy at the right side was modified into a *P1-rw*-like sequence as outlined in Figure 5 (see MULE, filler DNA and repeats depicted as purple, pink and light blue rectangles, respectively). The green rectangle stands for part of a sequence, which was shown to have enhancer function in *P1-rr*. A model explaining the conversion from a *P1-wr *and *P1-rw1077 *sequence to a unique *P1-rr *structure is briefly described. A DNA double-strand break of unknown cause was expanded by exonuclease activity. DNA repair occurred by non-homologous end-joining as evidenced by filler DNA (yellow rectangle). While the initial 9 bp (ATAATTGGG) of the filler DNA stem from a sequence 55 bp downstream of the deletion end point (see light blue balloon), the adjacent 4 bp TCAC correspond to a sequence 21 bp upstream of the insertion site (tan balloon). The MULE fragment contains an 8-bp sequence TCGATGCC also found 1269 bp further downstream (shown on top of the grey rectangle). Unequal crossover at the 8-bp site resulted in the duplication of the 1269-bp sequence in tandem fashion and addition of a fourth exon.

*P1-rr *is structurally more complex than *P1-rw1077 *and a single *P1-wr[B73] *gene. *P1-rw1077 *and *P1-rr *contain the same MULE fragments and filler DNA inserted in exactly the same sequence position. However, a 1.2-kb duplication in *P1-rr *that partially includes the fragmented MULE suggests that *P1-rr *is derived from *P1-rw1077*. This duplication results in the addition of a fourth exon, which is unique to *P1-rr*. A closer look at the *P1-rr *3' UTR may help to shed light on the evolution of the *P1-rr *enhancer region (see bottom panel of Figure 6).

Whereas *P1-rw1077 *and *P1-rr *are identical in the initial 3' UTR, they diverge 35 bp following the stop codon. The next 13 bp (ATAATTGGGTCAC) in *P1-rr *originated from two separated *P1-rw1077 *sequences, 1,410 bp apart, implying a deletion event in *P1-rr *compared to *P1-rw1077*. The 13-bp (ATAATTGGGTCAC) insertion in *P1-rr *can be assigned to *P1-rw1077 *sequences upstream and downstream of the deletion site. ATAATTGGG is duplicated 59 bp downstream and includes the first two bp of the MULE TIR. Obviously, the adjacent TCAC occurs frequently within the *P1-rw1077 *sequence. However, the closest TCAC can be located 21 bp upstream of the insertion site. The 13-bp *P1-rr *sequence subsequent of the point of divergence with *P1-rw1077 *is suggestive of filler DNA, indicating that a previous DNA double-strand break in *P1-rw1077 *was restored by the NHEJ pathway. A tandem duplication of 1,269 bp that comprises the majority of both MULE fragments and 3' flanking enhancer sequences generated the current *P1-rr *3' end and enhancer region.

In summary, DNA double-strand breaks in a *P1-wr*-like tandem array were probably repaired by NHEJ events that could have resulted in the rearrangements and duplications of enhancer-carrying sequences and consequently in novel *p1 *alleles as discussed below.

### The *p *alleles differ in their 3' UTRs and polyadenylation sites

Although *p1 *and *p2 *share nearly the same coding sequences, their downstream sequences vary remarkably (Additional file 5: Supplemental Figure S3). Sequence alignments of *p1 *and *p2 *alleles revealed that the *P1-rr4B2 *and *p2 *divergence from *P1-wr[B73] *is caused by transposon insertions. In *P1-rr4B2*, a *Mu*-like element was placed 109 bp downstream of the stop codon probably due to a deletion event (see above), and *p2 *alleles are followed by an *Eninu *retroelement 248 bp after the stop codon. The insertion sites close to the stop codon raise the question whether these transposable elements eliminated the transcription termination signals and the polyadenylation sites in the *P1-rr4B2 *and *p2 *3' UTRs. In general, the 3' UTR is also important for post-transcriptional regulation such as microRNAs and translational control, and gain or loss of *cis *elements within the 3' UTR could contribute to allelic diversity. Therefore, we decided to map the polyadenylation sites of these alleles.

The *P1-wr[B73] *coding sequence is not flanked by transposons, and its 3' UTR probably represents the original 3' UTR structure of all *p *alleles before transposon modifications. We performed 3' RACE experiments to identify the *P1-wr[B73] *3' UTR using three different gene-specific primers (p1 race 5'-1 to 3, see Table 1) and three independent pericarp tissue sources. RNA was extracted 20 days after pollination (DAP) from plants that contain the *P1-wr[B73] *cluster. Since the combination of different primers and tissues gave the same result we merged the data sets as shown in Figure 4A. Interestingly, we detected 18 polyadenylation sites in *P1-wr[B73] *that are spanning 189 nt from 141 to 329 nt after the stop codon. However, 36% of transcripts are polyadenylated 301 nt after the stop codon. The *P1-wr[B73] *cluster consists of 11 *P1-wr *tandem repeats and a *p2/p1[B73] *hybrid gene that differ by few polymorphisms in their transcribed regions. As these 18 polyadenylation sites are not specific for a particular repeat, the polymorphisms apparently do not affect the polyadenylation signals.

**Table 1:** PCR primers used in this report to amplify p2, p1 and flanking sequences and for 3' RACE.

p2-amplification	
p2-seq-2-for	CGCGTGATTGGCTCCTCGGATTACC

p2-seq-2-rev	TTTTCGGGACTGCGTGCATTGACTC

p2-seq-3-for	GGACGGCGGAGGAGGACCAGTTA

p2-seq-3-rev	TGATAGCTCGCCAGTTTTGTTAGAGGAT

p2-seq-4-for	ATGGCTGGCCCGATCGGTTGAGAGTTA

p2-seq-4-rev	CCGCTGCTGCTGTTGGGCTGGTTCG

p2-seq-6-for	CGCGCATTGGCTAGCTTCCCTGTT

p2-seq-6-rev	GCTTGTCGCCGGTCTCCATCTCCT

p2 3' RACE	

p2 race 5'-1	CTCCCGCCGGTGAAGTGAAGACAA

p2 race 5'-2	CGGACCGATCAGACAGACAGACAGACCA

p2 race 5'-3	GCCGTGGGTGCTGGAGCCGATAGA

p1 3' race	

p1 race 5'-1	GAGGAGGGGCCCAGCAGCGAGGAC

p1 race 5'-2	GCCGCCGAGCCGCTGGAAGTTGC

p1 race 5'-3	TCACCGGACCGATCAGACAGACCAACCA

3' flanking sequences	

shad-gene1-2 f	AGGGCAGCGTCTCCACCATCTA

shad-gene1-2 r	CAAAACCCTCAACCCCGTATTCTC

shad-gene1-3 f	CGTTGCTTCACTCCCCCGTTAGA

shad-gene1-3 r	GCTGATCAATGCGCTCGTCGTTC

r2-gene1-2a f	CGATGCATGCACTGTCCGATTTA

r2-gene1-2 r	CGGCGGTGGCGGCTACTTCT

r2-gene1-1 f	GCTACCCTCAATGCATGCACTGTCCT

r2-gene1-1 r	CGCGCTTCACGGGCTCACCAA

gene 1-2 f	GGACGAGCGGGACGAGGCGGTTAC

gene 1-2 r	GTCTGGCACTTCTTCCCCTGTCCT

RT-PCR results indicated that *p2[4Co 63]*, *p2[P1-rr4B2] *and *p1/p2[B73] *are expressed in silk tissue. Accordingly, we carried out 3' RACE experiments using total RNA from silk and three different primers (p2 race 5'-1 to 3, see Table 1), which hybridize to exon 3 of *p2*. The RNAs extracted from *p2[4Co63]*, *p2[P1-rr4B2] *and *p1/p2[B73] *lines produced almost identical results with all PCR primers, which allows us to combine the data for ease of presentation (Figure 4B). We found 19 polyadenylation sites in a 218-bp interval that is located between 139 and 356 nt past the *p2 *stop codon. Whereas seven minor polyadenylation sites (adding up to 21% of the total events) are upstream of the retrotransposon, 12 sites lie within the LTR, including the major site (33% of polyadenylated *p2 *mRNAs), which is 269 nt from the stop codon and 22 nt into the LTR. The sequence alignment between *p2 *and *P1-wr[B73] *shows that the main polyadenylation site of *P1-wr[B73] *is 87 bp past the point of *p2 *and *P1-wr[B73] *divergence. The equivalent *p2 *fragment was displaced by retroelement insertions, and therefore cannot serve its original function. Nevertheless, *p2 *was able to recruit alternative polyadenylation signals and sites located mostly in the *Eninu *LTR.

Subsequently, we performed 3' RACE experiments on *P1-rr4B2 *total RNA extracted from silk and one primer binding (p2 race 5'-3, see Table 1) to exon 3. This exon contains the 3' UTR of the alternatively spliced *P1-rr4B2 *transcript, which encodes the functional P protein. We sequenced significantly fewer clones compared to *P1-wr[B73] *and *p2 *and obtained fewer polyadenylation sites. While polyadenylation sites are distributed over 403 nt from 143 to 545 nt measured from stop codon, the first site is used most often (31%) (Figure 4C). All seven polyadenylation sites are located in the MULE fragments, two within the TIR, the remainder in the transcribed part. Due to the partial deletion of the former 3' UTR alternative polyadenylation signals and sites had to be employed from adjacent sequences as described above. Note that the MULE borders *P1-rr4B2 *in opposite transcriptional orientation. A transcript from an intact member of the same MULE family could therefore produce antisense RNA that is complementary to *P1-rr4B2 *mRNA.

## Discussion

### Models for the evolution of *p1 *alleles

A distinguishing feature of the *p *locus is its tremendous allelic diversity, which makes it a preferable locus to study evolutionary changes and chromosomal dynamics on a larger and smaller scale. Although the grass family arose by an ancient whole genome duplication (WGD) event [24], the *p *gene has only a single ortholog in rice and sorghum, indicating that one copy was lost from the paleoploid ancestral genome. However, the more recent allotetrapolidization event, which formed the ancestor of maize about 5 mya [9], gave rise to two *p *copies located in the homoeologous regions of chromosomes 9 and 1. The copy on chromosome 1 was then duplicated in tandem 2.75 mya, thereby evolving into the current *p2 *(ortholog) and *p1 *(paralog) genes [8,10]. The bulk of retrotranspositions in most grasses occurred more recently. A series of nested insertions that split approximately 80 bp of the *p *3' UTR occurred between 1.4 to 0.2 mya [10]. Although retroelements are highly repetitive in the genome, insertions of retroelements in a nested fashion create unique sequence junctions and become chromosomal markers [25]. However, we do not know whether the retroelements transposed into the paralog or ortholog repeat, or maybe even into a later-generated copy. A model proposed for the evolution of single-copy alleles states that the retroelement insertion occurred in the 3' UTR of *p2*, thereby separating *p1*, which turned into *P1-rr *and *P1-rw *[8,17] (Figure 3). In contrast, transposition into the 3' UTR of *p1 *retains the repeat structure and allows the amplification of additional copies by unequal crossover as suggested for the evolution of the multi-copy *P1-wr[B73] *allele [10] (Figure 3). Theoretically, only few recombination events are needed to transfer *p1 *and *p2 *sequences across the retroelement cluster. Therefore multi-copy alleles in a tandem array could have been created upstream, downstream or on both sides of the cluster simultaneously. These intermediate structures that enable us to discover the step-by-step evolution of all *p *alleles might still exist in the maize germplasm. Our current analysis allows us to present new and refined models for the evolution of *p1-ww[4Co63]*, *P1-rr4B2 *and *P1-rw1077*.

### Models for the evolution of *p1-ww[4Co63]*

*p1-ww *is a null allele because *p1 *specific sequences such as coding-, promoter-and proximal enhancer sequences are absent in 4Co63 (Figure 3). Is it possible that *p1-ww[4Co63] *represents a haplotype where the tandem duplication of the ancestral *p *gene never took place? According to this hypothesis, the nested retroelement insertions in 4Co63 that are identical to alleles containing *p1 *and *p2 *sequences must have happened before the *p *duplication event. However, since the *p *duplication occurred 2.75 mya, 1.37 million years before the first retroelement insertion, we can disregard this possibility [8,10]. Thus, the alternative explanation that a functional *p1 *allele was deleted to give rise to *p1-ww[Co63] *is more likely. The *p1-ww[4Co63] *structure does not reveal the functional *p1 *allele(s) and their deletion or recombination events that resulted in the current null allele. Considering that *p1 *alleles are located on both sides of the retroelement cluster, multiple recombination events could have occurred to create the *p1-ww[4Co63] *allele.

**Figure 7:**
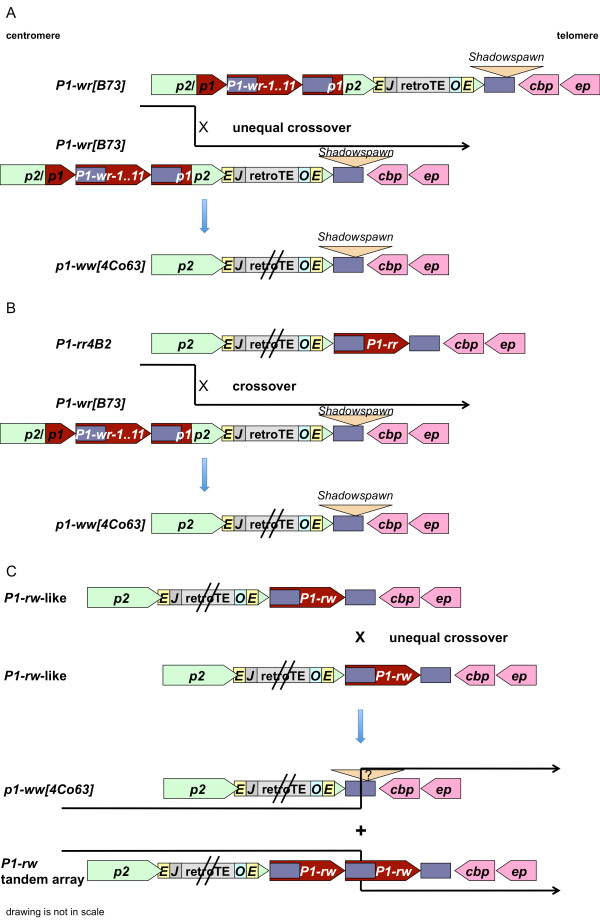
**Models for the origin of *p1-ww[4Co63]***. Various models may help explain the evolution of *p1-ww[4Co63]*. Notice that the same color code is used here as in Figure 3, which illustrates schematic alignment of *p1 *and *p2 *alleles. (A) Expansion and contraction of the *p *locus generates *P1-wr[B73] *and *p1-ww[4Co63]*, respectively. A simplified representation of the *P1-wr[B73] *cluster is shown. Unequal crossing over between *p2/p1[B73] *(or *p2*) and *p1/p2[B73] *could have caused the deletion of the internal *P1-wr[B73] *repeats. Such a recombination event could have occurred any time during the evolution of the cluster, independent of the *P1-wr *copy number, retroelement insertions at the 3' end and the deletion at the *p2*/*P1-wr *junction sequence. (B) Similarly, alignment along the retrotransposon cluster and crossover between *P1-rr *and *P1-wr[B73] *could have generated the *p1-ww[4Co63] *structure. (C) A misalignment at the repeat sequences of *P1-rw-*like alleles, which contained the simple *P1-wr[B73] *distal enhancer region, and unequal crossover could have led to a duplication or deletion event giving rise to *p1-ww[4Co63]*. However, this model does not account for the *Shadowspawn *retroelement in *p1-ww[4Co63]*.

One possible scenario for the origin of *p1-ww *in inbred line 4Co63 is that the null allele, which carries a functional *p2*, is derived from *P1-wr[B73]*. While unequal crossover among repeat sequences can lead to an increase of copy numbers, the alternative outcome is a reduction of repeats. During the evolution of the *P1-wr[B73] *allele, unequal crossover between the flanking genes of the cluster, namely *p2/p1[B73] *and *p1/p2[B73]*, could have caused the deletion of all *P1-wr[B73] *repeats, and would still have generated a functional *p2 *gene (Figure 7A). However, *p2 *in 4Co63 differs by various SNPs and indels from the corresponding *p2/p1[B73] *and *p1/p2[B73] *sequences of *P1-wr[B73]*, indicating that *P1-wr[B73] *might not be the immediate progenitor for *p1-ww[4Co63]*.

*p1-ww[4Co63] *also could have evolved by a recombination event that involved two different *p1 *alleles. Unequal crossing over between *p2 *of *P1-rr4B2 *and *p1/p2[B73] *of *P1-wr[B73] *could have generated the current *p1-ww[4Co63] *structure and could have restored the *p2 *copy (Figure 7B). Even then, the deletion of the original paralog would have been derived from the *P1-wr[B73] *allele. Nevertheless, this could not have happened recently (on an evolutionary time scale) because of sequence polymorphisms in the participating alleles. Interestingly, both *p1-ww[4Co63] *and *P1-wr[B73] *carry as a signature the *Shadowspawn *retroelement in the same position, indicating that *p1-ww[4Co63] *most likely derived from *P1-wr[B73] *in multiple steps.

In addition, we can envision a *P1-rw*-like allele, which is similar to *P1-wr[B73] *in the distal enhancer structure. Such a *P1-rw *allele has been described [17]. An unequal crossover between the large repeats flanking the coding regions duplicates the *p1 *gene or deletes the coding sequences, resulting in the *p1-ww[4Co63] *structure (Figure 7C). This scenario resembles the origin of *p1-ww1112 *[14]. However, this model does not directly account for the *Shadowspawn *retroelement in *p1-ww[4Co63]*. All models demonstrate the complexity of the *p *locus and reveal the countless possibilities for recombination to occur whenever paralogous sequences are present.

### Model for the evolution of *P1-rw1077 *and *P1-rr4B2*, with focus on regulatory sequences

Despite the repeat structure of the *P1-wr[B73] *cluster, a single *P1-wr[B73] *copy has the least complicated *p1 *allele composition, followed by *P1-rw1077 *and then *P1-rr4B2*. We hypothesize that *P1-rw1077 *originated from a *P1-wr-*like tandem array (Figure 5) because *P1-rw1077 *comprises a sequence fragment downstream of the *p2 *section that is virtually identical with the junction sequence of two *P1-wr[B73] *repeats in a head-to-tail assembly. This *P1-wr *tandem array could have been located on either side of the retroelement cluster.

A plausible sequence of events is as follows. A *Mu*-like element inserted into one of the *P1-wr *repeats 1,204 bp after exon 3 of the previous copy. Then an aberrant transposition event (abortive excision event) of this MULE caused a DNA double-strand break that enabled exonucleases to digest the unprotected DNA ends (Figure 5) thereby extending the gap into the adjacent *P1-wr *repeat. The deletion would have included MULE sequences (about 3.5 kb compared to a putative autonomous element) and almost the entire length of a *P1-wr *repeat (more than 12 kb). Non-homologous end-joining [21-23,26], copying a 9-bp sequence (AACCTATGT) that is located 27 bp downstream from the deletion endpoint, must have repaired the break. Due to the nature of tandem repeats, the large deletion described above results in small repeats of 203 bp that are flanking the MULE fragments. Interestingly, this duplication is part of a 1.2-kb sequence that contains the enhancer element of *P1-rr*.

A single *P1-wr-*like allele downstream of the retroelement cluster that is flanked by large repeats due to the retroelement insertion in *p2 *could have been converted into a tandem array by unequal crossover between the large repeats (Figure 7C). Gene conversion events then could have transferred the altered region that originated at the 3' large repeat to the 5' large repeat where the distal enhancer sequence functions [17]. Alternatively, *P1-rw1077 *arose from *P1-wr *repeats upstream of the retrotransposon cluster. The sequence 3' of this cluster, which corresponds to the 3' intergenic region of *p2 *as found in the *P1-wr[B73] *cluster and *p1-ww[4Co63]*, is nearly identical with the 5' end of a *P1-wr[B73] *repeat over a stretch of 5.2 kb (Figure 2 and Additional file 6: Supplemental Figure S4A). Due to this sequence similarity, a recombination event between *p1-ww[4Co63] *and the proposed *P1-rw1077 *precursor could have occurred that positioned *P1-rw1077 *downstream of the retrotransposon cluster (Additional file 6: Supplemental Figure S4A). This arrangement assumes that the *P1-rw1077 *allele resembles *p1-ww[4Co63] *at the 5' end. After the recombination break point, *P1-rw1077 *has to be closer to *P1-wr[B73] *because, based on our model, *P1-rw1077 *is derived from *P1-wr*. Indeed, a sufficient amount of polymorphisms between the *p1-ww[4Co63] *and *P1-wr[B73] *alleles enables us to verify the predicted structure and to place the possible recombination site between 567 and 713 bp after the point of *p1-ww[4Co63] *and *P1-wr[B73] *alignment. Further recombination/gene conversion events contributed to the evolution of the present *P1-rw1077 *allele.

The presence of the MULE fragments and filler DNA in *P1-rr *in exactly the same sequence context as in *P1-rw1077 *agrees with our model that *P1-rr *continued to evolve from *P1-rw1077*. In our model for the origin of *P1-rr*, we propose a second DNA double-strand break (DSB) that occurred in *P1-rw1077 *in between the stop codon and the MULE insertion (Figure 6). In contrast to the first DSB, there is no evidence for the participation of a TE, leaving the cause for the DSB unknown. Exonuclease activities expanded the gap until both ends were joined in a NHEJ fashion by synthesizing two short DNA pieces (filler DNAs) from sites close to the deletion end points into the repair site. The DSB repair caused a deletion of 1,410 bp across the repeat junction that spanned almost the entire sequence from the stop codon to the MULE fragments. Interestingly, this intermediate *P1-rr *structure can be found at the 3' end of *P1-rr1088*, *P1-rrCFS36 *and *P1-rwCFS342 *[17].

The 5' transposon fragment happened to contain an 8-bp sequence close to the TIR (55-62 bp) that is present 1,269 bp further downstream as well. Unequal crossover between those 8 bp resulted in a tandem direct duplication of this 1,269 bp sequence. Accordingly, the final 318 bp of exon three, being part of the repeat, were replicated, too. A sequence at the 3' end of the first repeat was adopted as a splice acceptor site thereby generating a fourth exon. Although alternative splicing of exon 1, 2 and 4 has been reported, the protein product is of unknown function or may not have any function at all [27].

This putative evolutionary pathway explains how the *P1-wr *3' UTR was almost entirely replaced by a MULE, how the fourth exon unique to *P1-rr *was generated and how the 1,269 bp SalI fragment containing the *P1-rr *distal enhancer was nearly completely duplicated (the initial 175 bp of the enhancer region are missing from the first repeat). Subsequently, gene conversion events could have placed part of the modified enhancer sequence of the downstream copy to the upstream large repeat [17]. Alternatively, if this *P1-rr *module arose on the *P1-wr[B73] *side of the retroelement cluster as we also discussed for *P1-rw1077*, then a recombination event between *p1-ww[4Co63] *and the *P1-rr *ancestor could have transferred the *P1-rr *end to a position downstream of the retrotransposon cluster (Additional file 6: Supplemental Figure S4B). The crossing over took place in the 595 bp sequence between the duplicated MULEs, which is why the repeat structure of *P1-rr *at the 5' end differs from the 3' end whereas they are identical in *P1-rw1077*. Lastly, a 1.6 kb *hAT*-like transposable element inserted 340 bp upstream of the MULE or 159 bp 5' of the enhancer region. This transposition did not occur in *P1-rr1088 *[17]. Taken together, the novel distal enhancer structure of *P1-rr *could be the result of a MULE insertion and excision, deletion and repair by NHEJ, and duplication and deletion by recombination. This series of events from *P1-wr *to *P1-rr *confirms the sequential model of *P1-rw *and *P1-rr *evolution based on phylogentic analysis [17].

### Function of the enhancer region rearrangements on *p1 *expression

When the *p1 *paralog was formed, it probably included the complete *p *coding sequence and the basal promoter that controls *p *expression in silk tissue. Then the paralog acquired two additional regulatory sequences adding equally to the basal expression in pericarp and glume. The enhancer sequences were identified and tested in transient and transgenic plants using *P1-rr *fragments fused to a GUS reporter gene [19,20]. A 1-kb sequence adjacent to the promoter contains a regulatory sequence termed proximal enhancer while a 1.2-kb fragment further upstream includes a distal enhancer (Figure 2). The proximal enhancer region corresponds mostly to a truncated MULE that captured part of a host gene in between the TIR [10]. The proximal enhancer region and the basal promoter sequence are virtually identical in all sequenced *p1 *alleles to date (Figure 2). In contrast, the distal enhancer region varies in all *p1 *alleles as described above. Therefore, we hypothesize that the different spatial and temporal expression patterns of *p1 *alleles are caused by distinct distal enhancer regions [17]. The distal enhancer as defined in *P1-rr *is located within a 1,269-bp *Sal*I fragment [19,20], out of which 671 bp are derived form the *Mu*-like transposon (Figure 2). Although this MULE fragment is missing in *P1-wr[B73]*, transgenes constructed from *P1-wr *upstream regulatory sequences linked to *P1-rr *cDNA produced red pericarp and cob glumes in transgenic plants [28], indicating that the enhancer sequence is included in the 589-bp region downstream of the MULE. Since this 589-bp region is duplicated in *P1-rr*, *P1-rr *has two enhancer sites that are separated by the MULE fragment. Additional *P1-rr *alleles, namely *P1-rr1088 *and *P1-rrCFS36*, were shown to have the same enhancer structure as *P1-rr4B2 *with exception of the missing *hAT *insertion in *P1-rr1088 *[17]. Therefore, the *hAT *transposable elements inserted in the upstream copy of the enhancer region of *P1-rr4B2 *and *P1-rrCFS36 *obviously do not disrupt the enhancer sequence and function. Compared to *P1-rr*, *P1-rw1077 *has a deletion of 381 bp in the upstream repeat, which causes the loss of cob glume pigmentation [13]. Interestingly, two additional *P1-rw *alleles, *P1-rwCFS302 *and *P1-rwCFS342*, lack the entire upstream repeat and the MULE fragment, thus having the identical enhancer arrangement as a single *P1-wr[B73] *copy [17]. Taken together, the analysis of three *P1-rr *and three *P1-rw *alleles revealed that *P1-rr *alleles contain two copies of the specific enhancer sequence while *P1-rw *alleles only have one [17]. Interestingly, this region coincides with a tissue-specific DNase I-hypersensitive site that remains closed in pericarp tissue of *P1-pr*, a silenced epiallele of *P1-rr4B *[29]; the *P1-pr *phenotype is shown in Figure 1A. It was reasoned that the upstream enhancer repeat that is missing in *P1-rw1077 *controls the glume-specific expression in a position-dependent manner, since the identical enhancer region is located 671 bp further downstream [13]. An alternative explanation was prompted by the fact that *p1 *expression in pericarp is weaker and delayed in *P1-rw1077 *compared to *P1-rr*. We hypothesize that the transcriptional strength of *p1 *alleles is correlated with the enhancer copy number, which is supported by similar findings in human upstream enhancers [30]. Consequently, *P1-rw1077 *produces less P1 protein than *P1-rr *in all expressing tissues. Also, each *p1 *allele is not expressed uniformly in female and male floral tissues within a plant. For example in *P1-rr*, *p1 *transcription is usually higher in pericarp than in cob glumes [15].

Therefore, we propose that the presence of only one distal enhancer site in the *P1-rw1077 *allele results in weak expression in pericarp tissue but no expression in cob glumes. Due to the duplication of the enhancer sequence as outlined in our model, *p1 *transcription in pericarp and glume tissue was equally elevated such that *p1 *is strongly expressed in pericarp and weakly expressed in glumes, thereby giving rise to *P1-rr *alleles. Note that comparisons with *P1-wr *alleles are not appropriate due to their post-transcriptional silencing, which potentially is repeat induced [31]. This model is supported by an analysis of the spatial expression pattern in transgenic plants where various *p1 *constructs were expressed only in few *p *expressing tissues, resembling *P1-rr *or *P1-rw *phenotypes. It was shown in these transgenic plants that *p1 *expression follows a spatial hierarchy that begins with pericarp and continues with cob glumes, husk, silk, and tassel glumes in decreasing order [32,33]. For instance, if the transgenes had been expressed in only one tissue, then it would have had to be in pericarp, in the case of two tissues then in pericarp and glumes, and so on.

### The *p *alleles differ in their 3' UTR

Polyadenylation is involved in many facets of mRNA metabolism including enhancement of mRNA stability, transport of mRNA from the nucleus into the cytoplasm, and regulation of mRNA translation. Although polyadenylation signals in plants are less conserved than in mammals [34], three signals were identified in maize, rice, and Arabidopsis: the far upstream element (FUE, located -150 to -35 nt upstream of the cleavage site), the near upstream element (NUE, situated -35 to -10 nt upstream of the cleavage site) and the cleavage element (CE, positioned -10 to +15 nt upstream and downstreams of the cleavage site) [35,36]. As we have shown above, a fragmented MULE was placed adjacent to the *P1-rr4B2 *stop codon possibly due to a NHEJ event. All mapped polyadenlation sites of the *P1-rr4B2 *transcript are located within the MULE sequence, indicating that *P1-rr4B2 *successfully recruited alternative polyadenylation signals in the transposon. Similarly, a *Mu *insertion in the 3' UTR of the *rf2a *locus also resulted in the adoption of new polyadenylation signals and sites [37]. Retroelements, the most common transposons in maize, also insert in 3' UTRs without disrupting polyadenylation as demonstrated above for the *p2 *alleles. Our results suggest that polyadenylation in maize is a highly dynamic process which despite its importance for the cell is not tightly regulated. The large amount of polyadenylation sites found in our analysis of *P1-wr[B73] *transcripts that do not contain a transposon insertion supports this conclusion. A genome-wide analysis of genomic and transcript data could shed light on the mechanism of polyadenylation in maize and could establish the proportion of genes that terminate in transposable elements. Interestingly, it has been shown that many polyadenlylation signals in human and mouse genes have been derived from transposable elements [38]. Besides polyadenylation signals, transcriptional as well as translational regulators have been identified in the 3' UTR of plant and animal genes, and their gain or loss could cause allelic diversity. For example, targets of microRNAs are often located in 3' UTRs [38,39].

### Gene copying events promote allelic diversity

Recombination is crucial for the evolution of genomes [40,41]. In particular, the non-homologous recombination pathway is frequently used to repair DNA double-strand breaks in somatic plant cells [26]. Previously, we reported a probable NHEJ event involved in the formation of the *P1-wr[B73] *cluster [10] that produced a hybrid gene due to the ligation of deletion end points located within two genes. Similarly, deletions and repair by NHEJ in the above mentioned alleles could have resulted in the restructuring of an enhancer region and formation of a novel 3' UTR.

The exceptional allelic variation at the *p *locus prompts the question about its similarities and differences to genes that exhibit less variation. We propose that the main cause for the diversity might lie in tandem gene amplification [8,17,42,43]. Once a gene underwent an initial tandem duplication, multiple unequal recombination events can follow as seen in the *P1-wr[B73] *multi-gene cluster [10]. A single crossing over or gene conversion event between misaligned paralogous gene copies can generate many new alleles including deletion and amplification derivatives. Interestingly, in plants such events can occur mitotically and can be transmitted into the next generation, thereby increasing allelic variation [44]. This explanation then implies that other loci exhibiting an increased allelic variation are multi-copy genes as well. Indeed, the complex *r1 *locus in maize is analogous to *p1 *in many aspects. The *r1 *locus, which also encodes a transcription factor, confers bluish anthocyanin pigmentation to various vegetative and floral plant tissues. Two *r1 *alleles, *R-st *and *R-r*, are molecularly well characterized. *R-st *contains various *r1 *genes, four of which are in tandem orientation [45]. *R-r *consists of one complete and three truncated *r1 *genes that originated from tandem duplication [46,47]. Comparable to *p1 *in complexity, both alleles undergo recombination and transposition events creating numerous derivative alleles. Paralogous gene copies in maize were also found at the *pl1 *[48] and *a1 *loci [49]. Especially the prolamine gene family with nearly 50 copies distributed over several chromosomes exemplifies the outcome of gene duplications [50]. Actually, a large proportion of genes are tandemly duplicated in Arabidopsis, rice, and maize [51-53]. Considering the amount of paralogous sequences and their possibilities to recombine, a single reference genome providing just one allele can obviously not reflect this allelic potential of the maize genome. Not surprisingly, a recent genomic comparison between the B73 and Mo17 inbred lines [54] revealed a large quantity of copy number variations and presence/absence variations confirming previous results [55]. Nonetheless, epialleles remain invisible in a traditional sequence comparison. Allelic diversity studies as presented here are essential for our understanding of the remarkably dynamic maize genome.

## Conclusion

Allelic diversity is the source for evolution and domestication. While allelic variation in wild species ensures the best possible adaption to changing environmental conditions, humans have profited from allelic pools in crop plants by selecting phenotypic variations that best meet their needs. Alleles differ most often in small-scale nucleotide polymorphisms but also in large-scale sequence rearrangements. Maize has been shown to be a highly polymorphic species well suited to study genome dynamics and the underlying molecular mechanisms. In particular, the maize *p *locus with its well-established genetic history offers a tremendous amount of ancient allelic variations, some representing intermediate steps in large-scale sequence rearrangements. The tandemly duplicated *p1 *and *p2 *genes encode virtually identical Myb-like transcriptional activators, but only *p1 *controls the accumulation of reddish flavonoid pigments in maize female and male floral organs. Because all P1 proteins are almost identical, the phenotypic variation must be due to *p1 *regulation. Therefore, this locus represents an ideal example of how genomic rearrangements can contribute to novel regulatory elements.

Here, we used targeted genome sequencing to apply comparative genomics to the maize genome. Sequence alignments of orthologs and paralogs of different genotypes of a single genomic region allow us to reconstruct the repair of double strand breaks from transposition events within gene copies and their flanking regions. Such drastic invasions of new sequence elements in flanking regions result in the de novo creation of regulatory elements involved in the transcriptional and post-transcriptional regulation of gene expression that differentiate gene copies in their function. Interestingly, sequence chimerism in the 3' untranslated portion of the mRNA gave rise to multiple poly-A addition signals with similar strength, indicating a more relaxed sequence restriction of the 3' processing machinery than previously believed.

## Methods

### Plant material

Seeds containing *P1-rr4B2 *and *P1-rw1077 *alleles, which were introgressed in a 4Co63 background, were thankfully provided by Tom Peterson, Iowa State University. The inbred lines B73 and 4Co63 carrying *P1-wr *and *p1-ww *alleles, respectively, were obtained from the Maize Genetics Cooperation Stock Center (maizecoop.cropsci.uiuc.edu) collection. Traditionally, *p1 *alleles are classified and named according to their pericarp and cob glume pigmentation, implicating that phenotypically similar but structurally different alleles share the same name. In this report, we use the inbred line where the *p1 *allele was originally described in as additional allelic designation such as *P1-wr[B73] *and *p1-ww[4Co63]*. Similarly, the inbred line will be used as allele description for *p2*, for example *p2[4Co63]*. Whenever the *p2 *source is unknown, the name of the linked *p1 *allele will be added to *p2*, such as *p2[P1-rr4B2] *and *p2[P1-rw1077]*.

### *p1-ww[4Co63] *isolation and sequencing

The inbred line 4Co63 contains a *p1-ww *allele, according to the colorless pericarp and cob phenotype of 4Co63 ears. We constructed a size-restricted lambda library using a lambda DASH II/*Eco*RI vector kit (Agilent Technologies) and *Eco*RI-digested 4Co63 genomic DNA. The lambda library was screened by hybridizing filters with probe 15 [12], which is derived from a distal enhancer fragment of *P1-rr *and is unique to *p1 *alleles. Two positively hybridizing lambda clones were isolated and subcloned into pBluescript II SK+ vectors (Agilent Technologies). Insert size and both end sequences of each clone were determined and found identical. A transposon minilibrary (Finnzymes) of one clone was constructed according to the manufacturer's instructions. Sequencing was performed with the ABI PRISM BigDye Terminator Cycle Sequencing Ready Reaction kit and an ABI 3730 capillary sequencer (Applied BioSystems). Sequence assembly and analysis were carried out using Lasergene (DNAstar) programs. Sequence gaps were closed by primer walking.

### *p2 *amplification and sequencing

Genomic PCR was performed to amplify *p2 *alleles. PCR primers (see Table 1) (Figure 1B) were designed based on *p2 *sequences from *p2[p1-ww1112] *[8], *p2/p1[B73] *and *p1/p2[B73] *[10]. The PCR-amplified products were cloned into pGEM-T Easy vector (Promega). The individual clones were completely sequenced using primers that are spanning the entire repeat length (approximately one primer every 300 bp, primer sequences available upon request). The sequencing reactions were carried out with the ABI PRISM BigDye Terminator Cycle Sequencing Ready Reaction kit and analyzed on an ABI 3730 capillary sequencer (Applied BioSystems). The sequences were assembled and evaluated with the Lasergene software (DNAstar).

### Sequencing of *P1-rr *3' noncoding region and flanking genes

The majority of *P1-rr *sequence was determined in *P1-ovov1114 *(orange variegated pericarp and cob) that is derived from *P1-vv*. The *Ac *element of *P1-vv *located in the second intron excised and reinserted 161 bp further upstream in the opposite direction [56], still allowing a considerable amount of phlobaphene accumulation in pericarp and cob. Similarly, *P1-rr4B2 *is a *P1-rr *revertant that also originated from *P1-vv *by *Ac *excision. When not otherwise specified, we use *P1-rr *(without additional allele designation) to refer to functional *P1-rr *alleles that are derived from the same *P1-vv*. Two *Eco*RI fragments, isolated from *P1-ovov1114*, were cloned in lambda using two *Eco*RI recognition sites outside of *P1-ovov1114*. The third site was provided by the *Ac *transposon [18]. The 3' fragment of 14.5 kb was further divided in two plasmids, SA206 and PA103, which we gratefully received from Thomas Peterson. A transposon minilibrary of both plasmids (Finnzymes) was constructed as per the manufacturer's protocol. Clones were sequenced using transposon primers, ABI 3730 capillary sequencers, and the ABI PRISM BigDye Terminator Cycle Sequencing Ready Reaction kit (Applied BioSystems). Both plasmids contain 12418 bp non-overlapping *P1-rr *and 3' flanking sequences.

### Amplification and sequencing of *p1 *intergenic region and flanking genes

Genomic PCR was performed to amplify *p1 *intergenic region and flanking genes. PCR primers (see Table 1) were designed based on corresponding sequences from B73 [10] and *P1-ovov1114 *(this study). The PCR products were cloned, sequenced and analyzed as described above for *p2*.

### Amplification of 3' cDNA ends (3' RACE)

Total RNA was extracted from pericarp tissue 20 days after pollination and emerging silk with the RNeasy Plant Mini Kit (Qiagen). RNA was reverse-transcribed to cDNA using the GeneRacer Kit (Invitrogen) with the GeneRacer oligo(dT) primers. cDNA was PCR amplified with the GeneRacer 3' primer and a gene-specific primer (see Table 1). In general, 96 RT-PCR products per primer pair (but only 18 for *P1-rr4B2 *samples) were cloned into pGEM-T Easy vector (Promega) and sequenced with universal primers. DNA sequences were analyzed with Lasergene (DNAstar) software. Polyadenylation sites were only plotted in Figure 6A to 6C, when they occurred more than once.

### Sequence annotation and GenBank accession numbers

The maize sequences were manually annotated using homology searches in various GenBank databases with multiple BLAST programs [57]. The sequences were submitted to GenBank and were assigned following accession numbers: *p2[4Co63]*: HM454271, *p2[P1-rr4B2]*: HM454272, *p2[P1-rw1077]*: HM454273, *p1-ww[4Co63]*: HM454274, *p1-ww[4Co63] *3' flanking region: HM454275, *P1-ovov1114 *3' end: HM454276

## Authors' contributions

WG and JM conceived of the study. WG designed and carried out the experiments. WG analyzed the data. WG and JM wrote the paper. All authors read and approved the final manuscript.

## Supplementary Material

Additional file 1 **P protein alignment**. Supplemental Figure S1 and figure legend. Click here for file

Additional file 2 **MULE structure and location**. Supplemental Figure S2 and figure legend. Click here for file

Additional file 3 **Characterization of a *Mutator*-like transposable element**. Supplemental description and Supplemental Tables S1-S3. Click here for file

Additional file 4 **Mechanism of NHEJ**. Supplemental information. Click here for file

Additional file 5 ***p *3' UTR sequence alignment**. Supplemental Figure S3 and figure legend. Click here for file

Additional file 6 **Recombination between modified *P1-wr *repeats and *p1-ww[4Co63] *can place *p1 *sequences across the retrotransposon cluster**. Supplemental Figure S4 and figure legend. Click here for file
