# Supplementary material for: Divergence of gene regulation through chromosomal rearrangements
Source: BMC Genomics. 2010 Nov 30;11:678. doi: 10.1186/1471-2164-11-678 (PMC3014980; doi:10.1186/1471-2164-11-678)
Supplement: Additional file 1 — P protein alignment. Supplemental Figure S1 and figure legend. [file 1471-2164-11-678-S1.DOCX]

Additional file 1:

**Supplemental Figure S1. P protein alignment.**


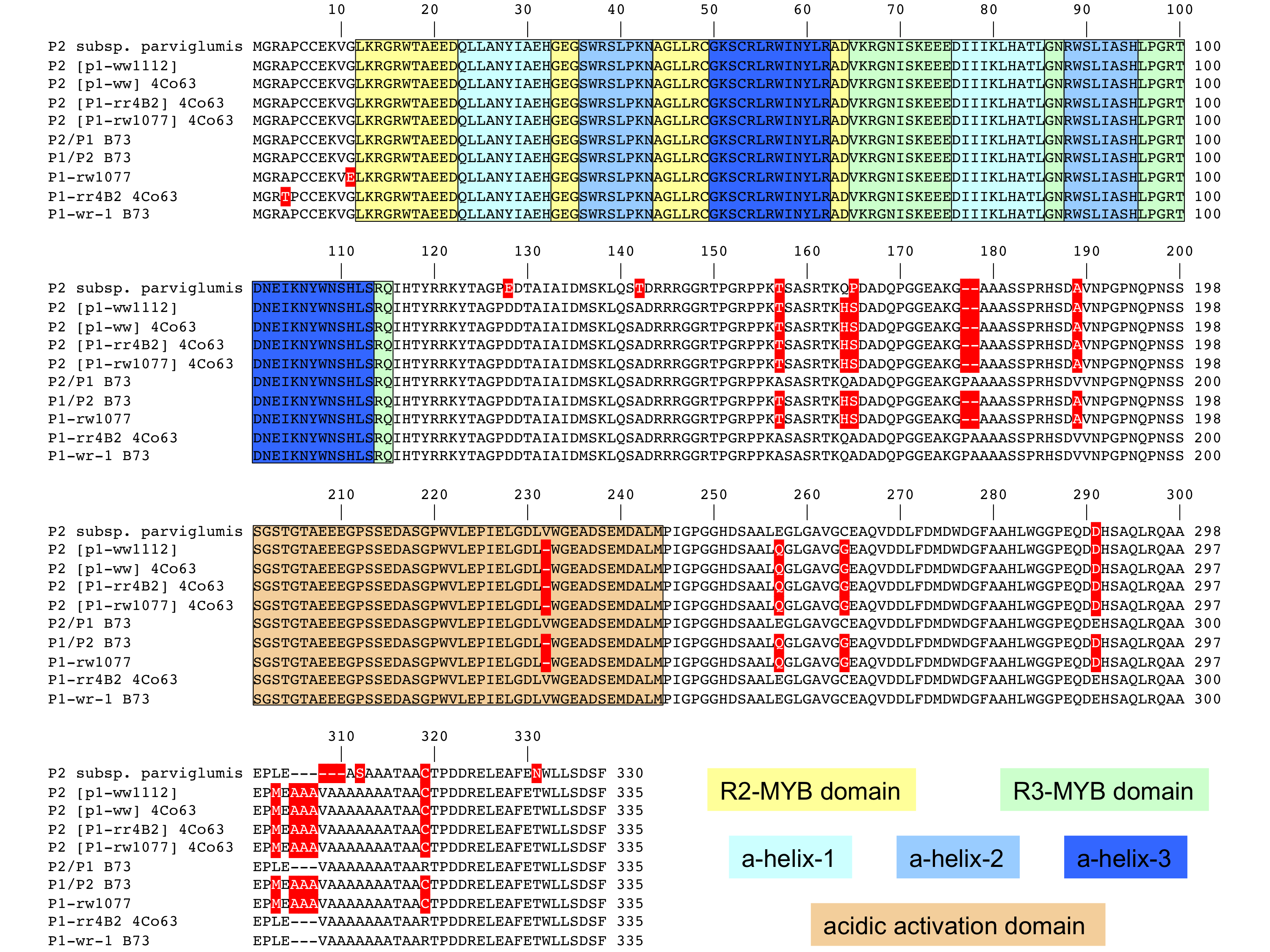


P1 protein sequences are highly similar to P2 and their chimeric P2/P1[B73] and P1/P2[B73] sequences. Maize inbred lines or subspecies name (*Zea mays* ssp. *parviglumis*) are given behind protein designations. Residues differing from P1-wr-1 B73 are written in white letters on red background. Functional P domains are framed. Notice that all proteins are identical in the R2 (yellow) and R3 (green) Myb domains. α-helices within Myb domains are shown in shades of blue. The acidic activation domain is boxed in tan color. GenBank accession numbers of sequences used to deduce the putative protein sequences are as follows: P2 subsp. parviglumis: AF210617, P2 [p1-ww1112]: AF210616, P2 [p1-ww] 4Co63: HM454271, P2 [P1-rr4B2] 4Co63: HM454272, P2 [P1-rw1077] 4Co63: HM454273, P2/P1 B73: FJ614806, P1/P2 B73: FJ614806, P1-rw1077: AY702552, P1-rr4B2 4Co63: AF427146 and HM454276, P1-wr-1 B73: FJ614806.
