# Supplementary material for: Divergence of gene regulation through chromosomal rearrangements
Source: BMC Genomics. 2010 Nov 30;11:678. doi: 10.1186/1471-2164-11-678 (PMC3014980; doi:10.1186/1471-2164-11-678)
Supplement: Additional file 2 — MULE structure and location. Supplemental Figure S2 and figure legend. [file 1471-2164-11-678-S2.DOCX]

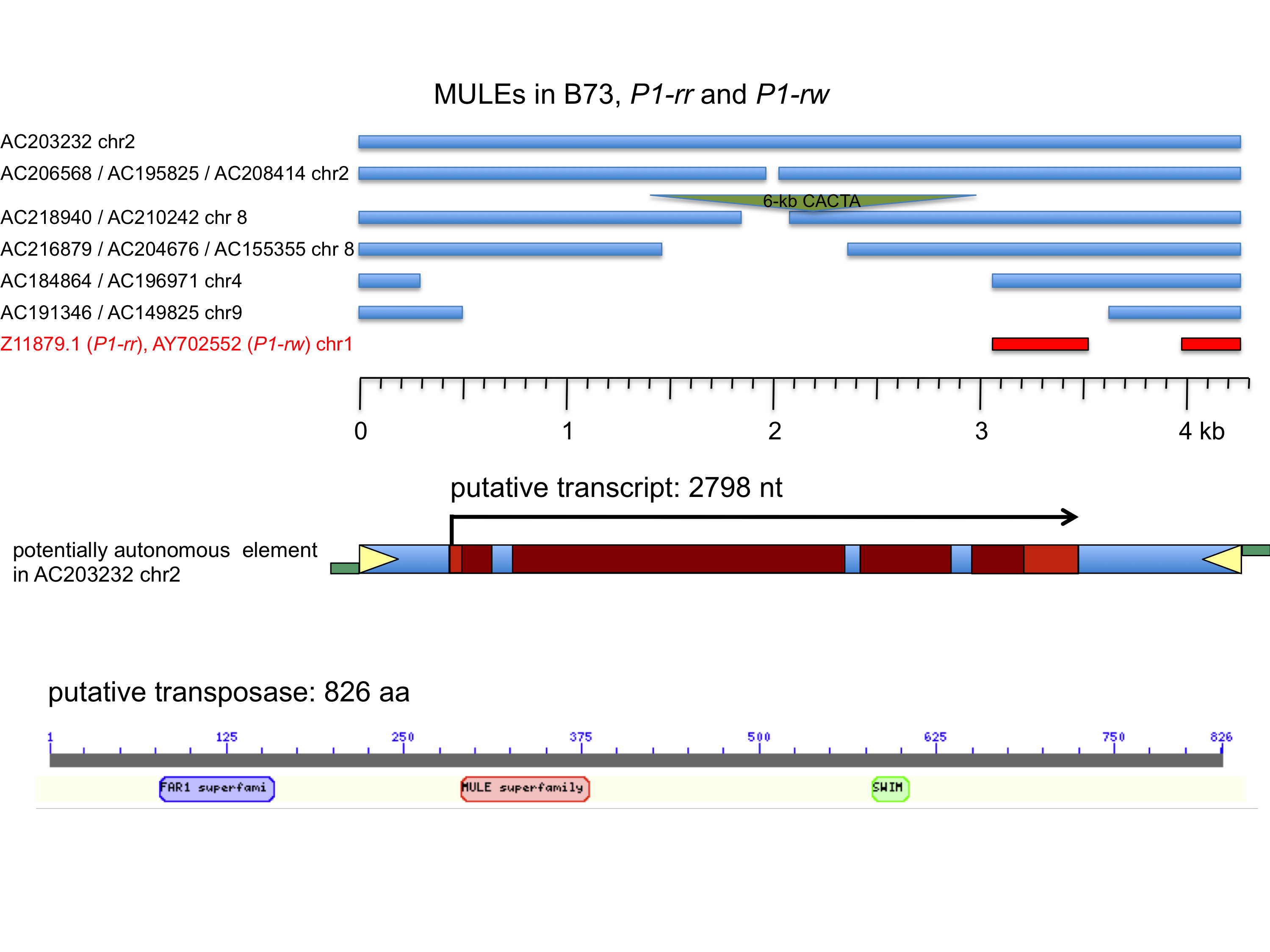
Additional file 2:

**Supplemental Figure S2. MULE structure and location.**

This previously uncharacterized MULE generates an 8-bp target site duplication upon insertion (green rectangles). It is delineated by 11-bp perfect terminal inverted repeats (TIR) or longer imperfect TIR (yellow triangles). A potentially autonomous element on chromosome 2 (accession AC203232) could produce a putative transcript of 2798 nt that could be translated into a transposase of 826 aa. Exons are depicted as dark red rectangles with lighter-colored UTRs. The putative transposase contains a FAR1, MULE and SWIM domain. Six copies were detected in the B73 genome, and their chromosomal location and sequence accession numbers are listed (two or more accession numbers merely indicate the redundancy of the BAC sequences). While most of the MULEs are truncated compared to the element in AC203232, the TE in AC218940 has a CACTA element inserted in its coding region. The fragmented element *in P1-rw1077 and P1-rr* on chromosome 1 is shown as red rectangles.
