# Supplementary material for: Divergence of gene regulation through chromosomal rearrangements
Source: BMC Genomics. 2010 Nov 30;11:678. doi: 10.1186/1471-2164-11-678 (PMC3014980; doi:10.1186/1471-2164-11-678)

Additional file 3:

**Characterization of a *Mutator*-like transposable element**

The fragmented transposon inserted in the 3’ UTR of *P1-rr* belongs to a family of *Mutator*-like transposable elements (MULEs) that terminate in 11-bp perfect repeats GGAAAAAAATT (Additional file 2: Supplemental Figure S2). Although the actual length of the terminal inverted repeats (TIRs) cannot be exactly determined due to the large amount of mismatches (Additional file 3: Supplemental Table S1), they are rather short compared to MULEs that are delineated by (mostly imperfect) TIRs of several hundred bp in size. BLASTN searches revealed that this family consists of six members in the B73 inbred line (Additional file 3: Supplemental table S1), therefore comprising a low-copy transposon family. One transposable element (TE) on chromosome 2 in B73 that is present in BAC clone ZMMBBc0548N08 (accession number AC203232) was analyzed as a prototype for this family. This element is 4258 bp long. Upon insertion, the element generates an 8-bp target site duplication (TSD), which is in the range of 8 to 10 bp characteristic for *Mu*-like elements. Based on fragmented maize EST evidence, a putative 2798-nt transcript could stem from this potentially autonomous element. This transcript encodes a deduced protein of 826 aa that contains a FAR1 DNA binding domain (pfam 03101), a MULE transposase domain (pfam 10551), and a SWIM zinc finger domain (pfam 04434), also characterized as ZnF PMZ (plant mutator transposase zinc finger domain, smart 00575) (Additional file 2). The most similar maize transposase carrying the same domains is derived from the *Jittery* MULE with 32% identities (49% positives) over a stretch of 584 aa.

The remaining five family members vary in size due to insertions and deletions. While four transposable elements are truncated compared to the prototype, one is significantly larger. It contains approximately half of a *Misfit* CACTA transposon inserted in opposite orientation at position 3104-7804. None of the elements encodes a functional transposase. They are inserted close to genes without interrupting coding sequences.

Interestingly, BLASTN searches using the above mentioned possibly functional transposase as query sequence reveal six additional MULE families in maize that differ in their TIR and transposase sequence (Additional file 3: Supplemental Table S2). Each family consists of two to four members in B73. Although every transposon family has largely distinct TIR they all share the initial sequence GGAAAAAAAT.

Most noticeably, the TIRs of these family members are more conserved and longer than the TIRs from the previously described family, ranging approximately from 85 to 164 bp. Only one MULE (within accession EF517601) out of 19 has the potential to encode a functional transposase of 681 aa, that has 65% identities (77% positives) with the aforementioned transposase over a core sequence of 671 aa. The smallest TE is 960 bp in size, the largest one contains several retrotransposons and measures 52239 bp. Whereas 8-bp TSDs are most frequent for these TEs, MULEs of one family generate a 9-bp TSD upon insertion.

However, another family was not discovered by similarity to transposase sequences because its elements only consist of TIRs. Two TEs previously identified in the *P1-wr[B73]* cluster (*p2/p1[B73]* and *P1-wr-8[B73]*) [10] are also delineated by GGAAAAAAATT sequences. About 60 additional copies, which measure roughly 200 bp each, can be detected in the B73 inbred line, demonstrating that this family expanded more than related ones. Nevertheless, no element in B73 has the capacity to encode a functional transposase.

Taken together, these eight MULE families constitute a superfamily in maize based on their identical flanking GGAAAAAAAT sequences and the similarities among their transposases.

TBLASTN searches using the maize transposase as query identified related MULEs in other grasses (Additional file 3: Supplemental Table S3). *Sorghum bicolor* cultivar BTx623 contains a family of four members that generate 9-bp TSDs upon insertion. The longest TIR in this family is 142 bp and the initial sequence GGAAGAAAATT varies in one bp from the maize TIR. Two members encode potentially functional transposases of 596 aa that are similar to maize (Identities = 386/515 (74%), Positives = 448/515 (86%), Gaps = 8/515 (1%)). The *Oryza sativa* subsp. *japonica* genome also contains this MULE family delineated by GGAAAAAAAT. Most of these rice elements are almost certainly non-autonomous although one TE in accession AL606101 carries a possibly functional transposase of 790 aa similar to maize (Identities = 506/781 (64%), Positives = 607/781 (77%), Gaps = 19/781 (2%)). Comparable MULE elements have been found in Brachypodium as well.

In summary, these low-copy MULE families are evolutionary conserved in grasses such as maize, sorghum, rice and Brachypodium. The mostly non-autonomous TEs are characterized by their 8- or 9-bp TSD, their initial TIR GGAAa/gAAAAT sequence, and their transposase similarities. These features imply that transposition requires the interaction of the conserved transposases with the GGAAa/gAAAAT binding site.


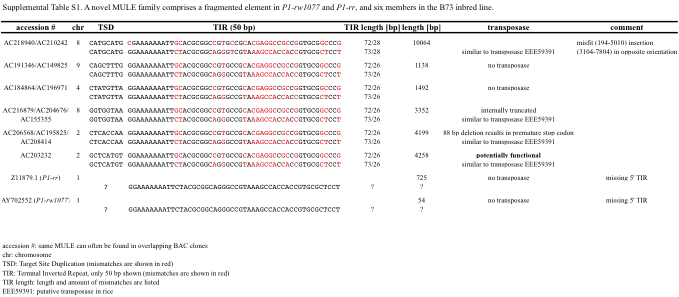


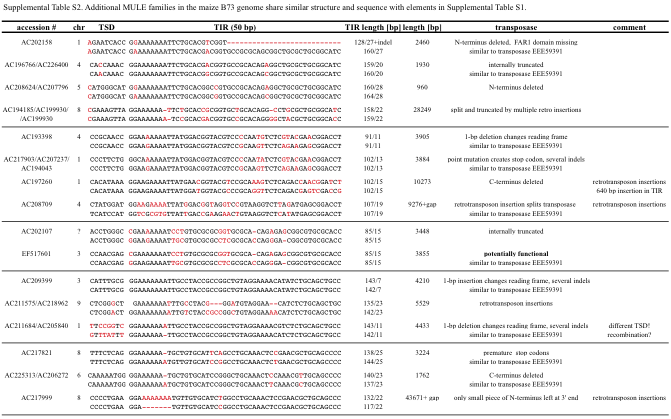


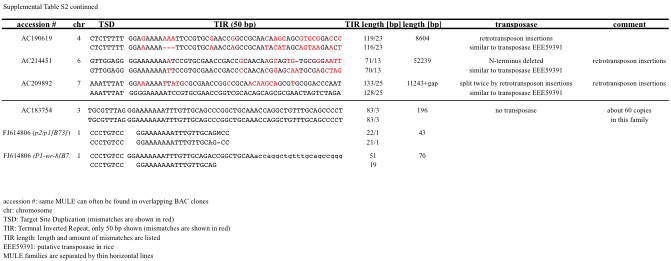


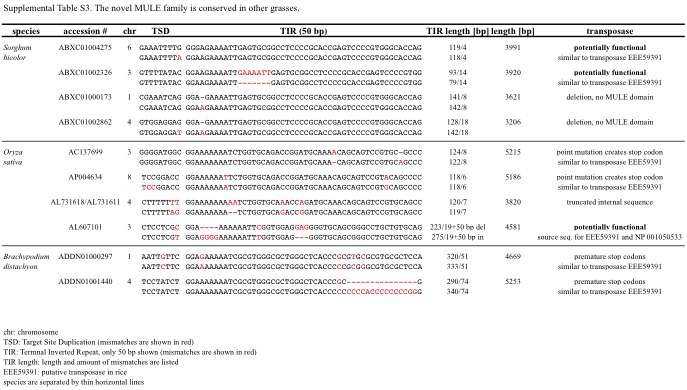

Supplement: Additional file 3 — Characterization of a Mutator-like transposable element. Supplemental description and Supplemental Tables S1-S3. [file 1471-2164-11-678-S3.DOCX]
