# Supplementary material for: Divergence of gene regulation through chromosomal rearrangements
Source: BMC Genomics. 2010 Nov 30;11:678. doi: 10.1186/1471-2164-11-678 (PMC3014980; doi:10.1186/1471-2164-11-678)
Supplement: Additional file 4 — Mechanism of NHEJ. Supplemental information. [file 1471-2164-11-678-S4.DOCX]

Additional file 4:

**Mechanism of NHEJ**

Many models have been proposed trying to explain NHEJ. The most appealing model is a combination of the single-strand annealing (SSA) model and the synthesis-dependent strand annealing (SDSA) model, since it can be also applied to illustrate mitotic homologous recombination [26]. The SSA-like mechanism states that after the DSB occurs, exonuclease activity produces 3’ single-stranded ends. Annealing of the single-stranded ends can happen at complementary sequences. In case of NHEJ, sites of microhomology of a few base pairs are sufficient for the annealing process, whereas for (intrachromosomal) homologous recombination the single-stranded sequences are required to be complementary over a larger region. Overhanging ends have to be removed before end ligation can take place. Single stranded regions are filled by DNA synthesis to complete the repair. This repair mechanism results in the deletion of sequences between the sites of homology and one out of two homologous sequences. However, an SSA-like mechanism cannot explain the presence of filler DNA at the deletion end points. The formation of filler DNA requires DNA synthesis from a template.

Like in the SSA-mechanism, the first step of the SDSA pathway suggests the generation of 3’ single-stranded ends via exonuclease digestion. Then one free 3’ single-strand invades a donor sequence by forming a D-loop and primes DNA synthesis. The acceptor molecule is elongated in a migrating replication bubble. The outcome of this repair process depends on the template. If a non-homologous template is invaded, filler DNA is produced. A template switch during this copying event can account for the origin of complex filler DNA. However, when the newly synthesized DNA is released from the replication bubble, single-strand annealing and ligation occurs by an SSA-like mechanism to finish the DSB repair. A similar pathway including slip mispairing during DNA repair synthesis is described for the formation of *Ds* derivatives following *Ac* excision. In case the free 3’ single-stranded terminus invades a homologous template, the outcome is gene conversion.
