# Supplementary material for: Divergence of gene regulation through chromosomal rearrangements
Source: BMC Genomics. 2010 Nov 30;11:678. doi: 10.1186/1471-2164-11-678 (PMC3014980; doi:10.1186/1471-2164-11-678)
Supplement: Additional file 5 — p 3' UTR sequence alignment. Supplemental Figure S3 and figure legend. [file 1471-2164-11-678-S5.DOCX]

Additional file 5:

**Supplemental Figure S3*
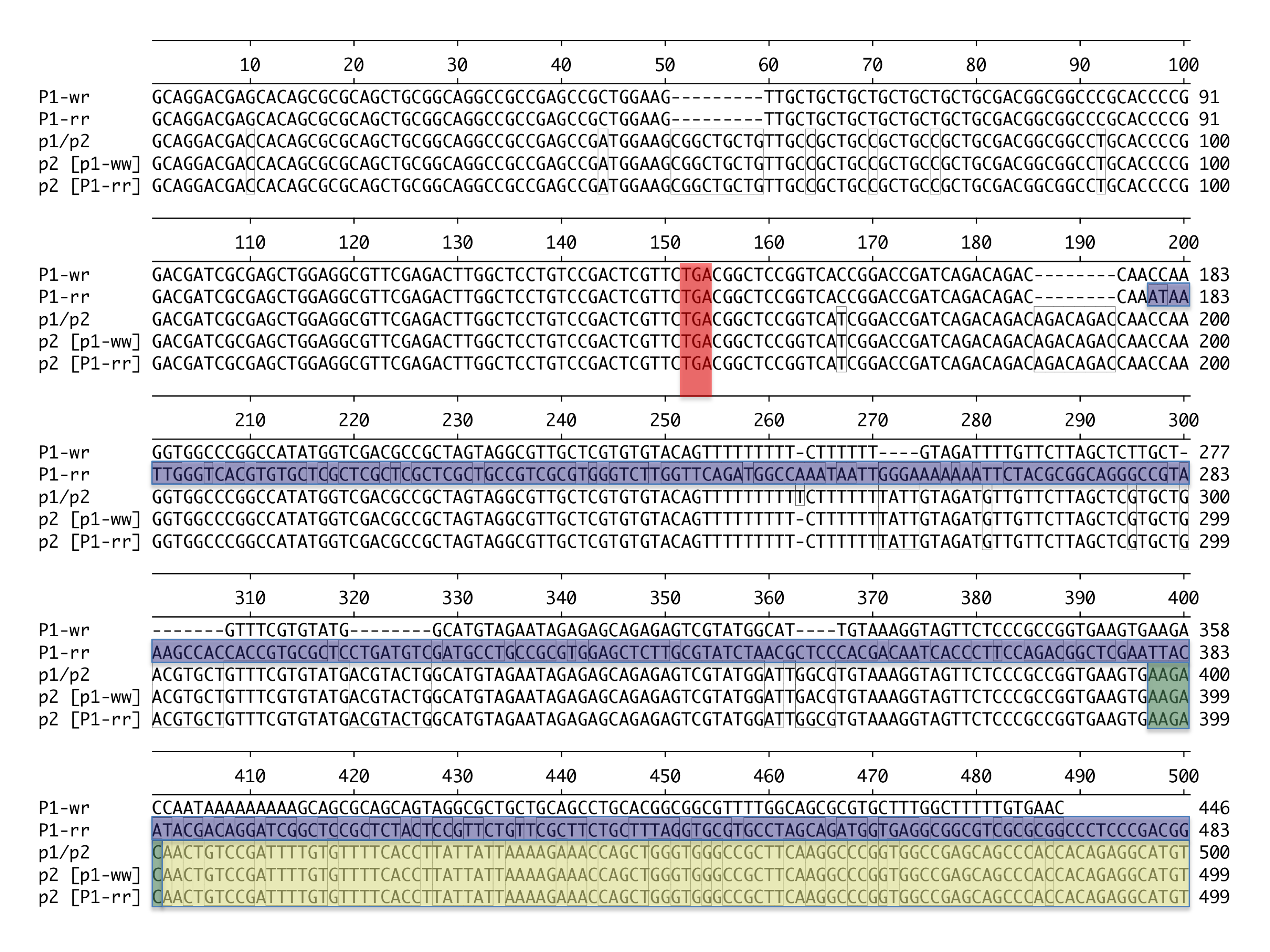
*. *p* 3’ UTR sequence alignment.**

Sequence alignments reveal a modified 3’ end of *P1-rr* and *p2* alleles compared to *P1-wr[B73].* Two representative *p2* alleles and the hybrid *p1/p2[B73]* gene are shown. Sequence polymorphisms are framed. The stop codon is boxed in red. Sequences after the point of divergence with *P1-wr[B73]* are shaded in purple for *P1-rr4B2* and shaded in yellow for *p2* alleles and *p1/p2[B73]*. The sequence highlighted in yellow is part of an *Eninu* retrotransposon. Note that the initial bp of *Eninu* are mutated from TG to AA. The target site duplication AAGAC is highlighted in green.
