# Supplementary material for: Divergence of gene regulation through chromosomal rearrangements
Source: BMC Genomics. 2010 Nov 30;11:678. doi: 10.1186/1471-2164-11-678 (PMC3014980; doi:10.1186/1471-2164-11-678)
Supplement: Additional file 6 — Recombination between modified P1-wr repeats and p1-ww[4Co63] can place p1 sequences across the retrotransposon cluster. Supplemental Figure S4 and figure legend. [file 1471-2164-11-678-S6.DOCX]

Additional file 6:

**Supplemental Figure S4. Recombination between modified *P1-wr* repeats and *p1-ww[4Co63]* can place *p1* sequences across the retrotransposon cluster.**

**
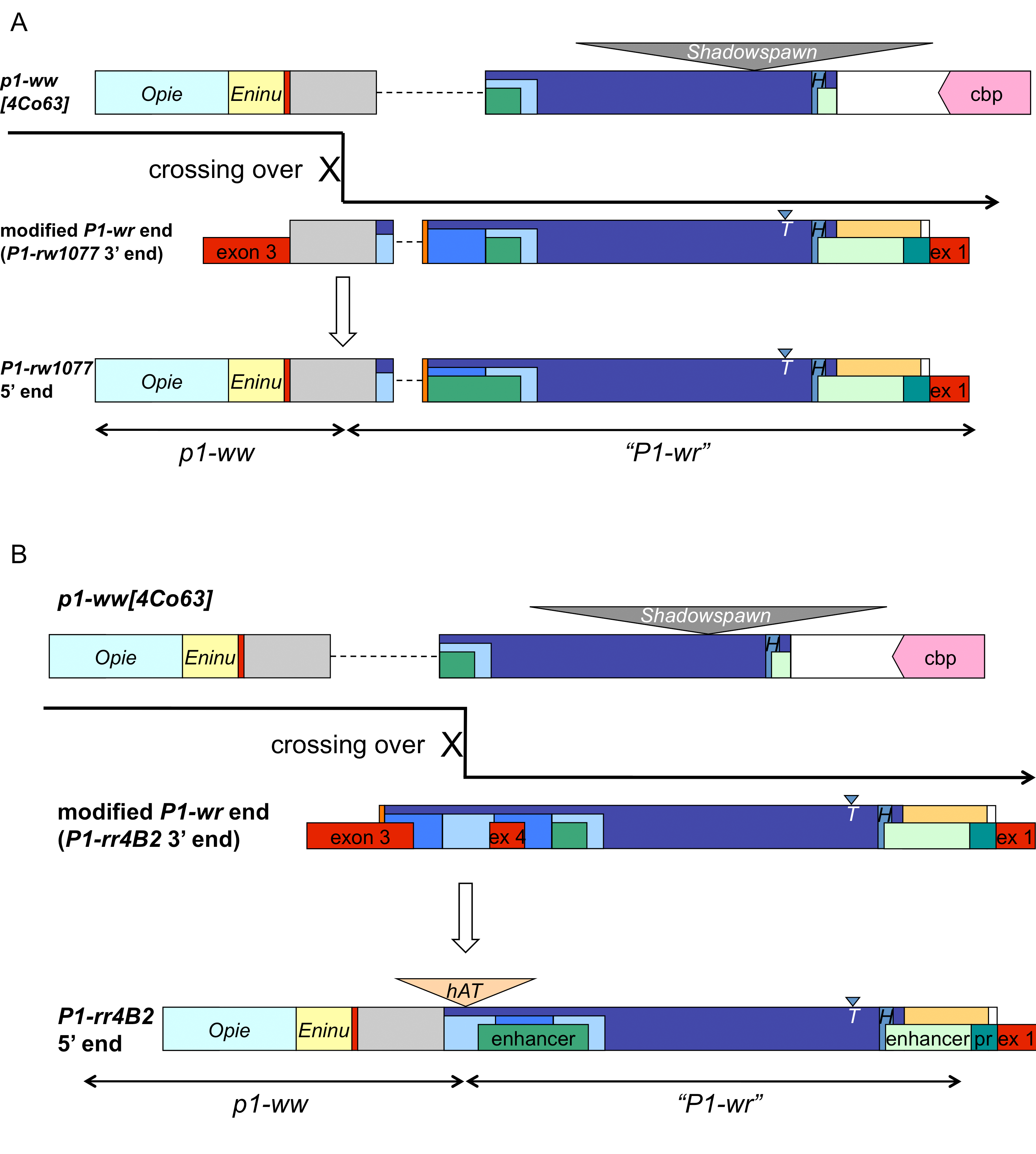
**

*P1-rw1077* and *P1-rr* could have originated within the *P1-wr* cluster, which is located upstream of the retrotransposon cluster. This model would require a recombination event to transfer the modified *P1-wr* sequences across the retroelement array. Subsequently, a second recombination step is necessary to shape the 3’ end of the novel *p1* allele. Alternatively, gene conversion events may have copied *p1* sequences downstream of the retroelement cluster.

(A) *P1-rw1077* 5’ end formation.

(B) *P1-rr* 5’ end formation.
